# Enhanced Ferroelectric Polarization in Au@BaTiO_3_ Yolk‐in‐Shell Nanostructure for Synergistic Boosting Visible‐Light‐ Piezocatalytic CO_2_ Reduction

**DOI:** 10.1002/advs.202410357

**Published:** 2024-10-16

**Authors:** Jun Hu, Rufang Zhao, Jingren Ni, Wei Luo, Hongjian Yu, Hongwei Huang, Boyuan Wu, Yang Wang, Jie Han, Rong Guo

**Affiliations:** ^1^ School of Chemistry and Chemical Engineering Yangzhou University Yangzhou 225002 China; ^2^ Hubei Key Laboratory of Pollutant Analysis & Reuse Technology College of Chemistry and Chemical Engineering Hubei Normal University Huangshi Hubei 435002 China; ^3^ Hubei Key Laboratory of Hydropower Engineering Construction and Management and College of Hydraulic & Environmental Engineering China Three Gorges University Yichang Hubei 443002 China; ^4^ Engineering Research Center of Ministry of Education for Geological Carbon Storage and Low Carbon Utilization of Resources Beijing Key Laboratory of Materials Utilization of Nonmetallic Minerals and Solid Wastes National Laboratory of Mineral Materials School of Material Sciences and Technology China University of Geosciences (Beijing) Beijing 100083 China; ^5^ Department of Physics The Chinese University of Hong Kong Shatin Hong Kong SAR 999077 China

**Keywords:** Au@BaTiO_3_ yolk‐in‐shell nanostructure, coupling effect, ferroelectric spontaneous polarization, visible‐light‐piezocatalytic CO_2_ reduction

## Abstract

Developing efficient photo‐piezocatalytic systems to achieve the conversion of renewable energy to chemical energy emerges enormous potential. However, poor catalytic efficiency remains a significant obstacle to future practical applications. Herein, a series of unique Au@BaTiO_3_ (Au@BT) yolk‐shell nanostructure photo‐piezocatalyst is constructed with single Au nanoparticle (Au NP) embedded in different positions within ferroelectric BaTiO_3_ hollow nanosphere (BT‐HNS). This special structure showcases excellent mechanical force sensitivity and provides ample plasmon‐induced interfacial charge‐transfer pathways. In addition, the powerful piezoelectric polarization electric field induced by the enhanced ferroelectric polarization electric field via corona poling treatment in BT‐HNS further promotes charge separation, CO_2_ adsorption and key intermediate conversion. Notably, BT with single Au NP encapsulated into hollow nanosphere shell with reinforced polarization (Au@BT‐1‐P) shows synergistically improved photo‐piezocatalytic CO_2_ reduction activity for producing CO with a high production rate of 31.29 µmol g^−1^ h^−1^ under visible light irradiation and ultrasonic vibration. This work highlights a generic tactic for optimized design of high‐performance and multifunctional nanostructured photo‐piezocatalyst. Meanwhile, these yolk‐in‐shell nanostructures with single Au nanoparticle as an ideal model may hold great promise to inspire in‐depth exploration of carrier dynamics and mechanistic understanding of the catalytic reaction.

## Graphical abstract

Yolk‐in‐shell nanostructured plasmonic hybrid photo‐piezocatalyst, with excellent mechanical force response, enhanced polarized electric field, and ample plasmon‐induced interfacial charge‐transfer pathways, exhibits exceptional synergistic visible‐light‐piezocatalytic CO_2_ reduction performance. Theoretical results reveal that the enhancement of ferroelectric polarization electric field not only serves as powerful driving force promoting carrier migration, but also lowers the energy barrier of forming the key intermediates.

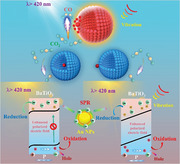

## 1 Introduction

The depletion of fossil energy sources and the growing concern over environmental pollution have ignited a pressing need for clean and sustainable energy solutions.^[^ ^1^, ^2^ ^]^ Among different alternatives, solar energy has gained widespread application in various fields such as photoelectric conversion,^[^ ^3^ ^]^ photothermal conversion,^[^ ^4^ ^]^ photoluminescence,^[^ ^5^ ^]^ and photocatalysis,^[^ ^6^ ^]^ which holds remarkable advantages lying in its cleanliness, cost‐effectiveness, and virtually unlimited supply. Notably, photocatalytic technology has been regarded as the most promising method to convert solar energy into chemical energy.^[^ ^7^ ^]^ However, the potential utilization always suffers from low catalytic activity due to the narrow photoabsorption and insufficient charge separation efficiency.^[^ ^8^ ^]^ Recently, plasmonic metal nanoparticles (NPs) that can perform outstanding charge‐carrier‐mediated reactions even under lower‐intensity visible light irradiation, have been regarded as effective candidates for overcoming critical challenges in heterogeneous catalysis.^[^ ^9^, ^10^, ^11^ ^]^ However, the hot carriers excited by localized surface plasmon resonance (LSPR) phenomenon will speedily decay radiatively or non‐radiatively on a femtosecond (fs) timescale, which immensely limits the opportunities for useful physical or chemical processes.^[^ ^12^ ^]^ Therefore, coupling with wide‐bandgap semiconductors to obtain various types of plasmonic nanometals/semiconductor composites photocatalysts can effectively prolong the lifetime of hot electrons.^[^ ^13^, ^14^ ^]^ Notwithstanding, deficient interfacial interaction and structural instability as critical factors remarkably impede their application and development in catalysis.

Yolk‐shell nanostructures, holding a hollow shell with high surface area and high‐activity interior core, have lately shown enormous potential on energy storage, biosensor, and catalysis.^[^ ^15^, ^16^ ^]^ Especially, functional NPs as active yolk are uniformly encapsulated in exterior hollow shells to avoid aggregation and inactivation thereby achieving potential applications of versatility.^[^ ^17^ ^]^ Migrating the movable yolk into the shell can introduce more interface interaction,^[^ ^18^ ^]^ moreover, this unique structure with the fixed active sites dedicates numerous advantages in catalysis, including enhanced activity, improved stability, optimized reaction conditions, and the ability to perform multiple functions simultaneously.^[^ ^19^, ^20^ ^]^ Our group has developed a template method to fabricate different yolk‐shell nanostructured catalysts, where a single Au NP as the “yolk” is encapsulated in each TiO_2_ hollow nanosphere shell or cavity.^[^ ^21^ ^]^ Compared with the structure of Au@TiO_2_ yolk‐shell and pure TiO_2_ nanocavity, the unique Au@TiO_2_ yolk‐in‐shell nanocatalyst shows much higher CO production (0.75 mmol h^−1^ g^−1^) and H_2_ production (95.6 mmol h^−1^ g^−1^) under visible light irradiation. The advanced catalytic performance is attributed to the intimate integration of the yolk and shell in this unique structure, which enhances the SPR of Au NP and accelerates the hot electron transfer from Au to TiO_2_. Despite these promising results, there is a growing need for functional breakthroughs in multi‐selective semiconductor shell. Therefore, developing effective strategies to promote the hot charge separation and transfer of metals and further elaborate the structural and multifunctional advantages of hollow spherical semiconductor shells is of great imperative.

The introduction of built‐in electric field has recently attracted broad attention to advance photocatalytic performance owing to affording a driving force to accelerate photogenerated charge transfer of photocatalysts,^[^ ^22^ ^]^ actually equated with a localized electrochemical process (i.e., the voltage is provided by space charge region).^[^ ^23^ ^]^ Ferroelectric semiconductors, coupling the piezoelectric effect and optical properties, open up a potential prospect to achieve a breakthrough in catalytic conversion efficiency through the collaboration of mechanical energy and solar energy based on piezotronics and piezo‐phototronics proposed by Wang's group.^[^ ^24^, ^25^ ^]^ BT as one of the earliest ABO_3_ compounds is one of the most famous representatives of perovskites with remarkable piezoelectric, ferroelectric, dielectric and electro‐optical properties applied in several major technology areas.^[^ ^26^ ^]^ In addition, BT has exhibited excellent photocatalytic and piezocatalytic performance across organic pollutants degradation, water splitting, N_2_ fixation, and CO_2_ reduction.^[^ ^27^ ^]^ Zhao et al. reported that BT, introducing optimized oxygen vacancies (Ov) concentration via NaBH_4_ thermal reduction, shows remarkable and stable photocatalytic activity toward N_2_ fixation with an NH_3_ yield rate of 1.35 mg L^−1^ h^−1^.^[^ ^28^ ^]^ In addition, the photocatalytic NH_3_ evolution activity can be further increased to 1.93 mg L^−1^ h^−1^ under a magnetic field. The experimental analysis and theoretical results highlight the crucial roles of the internal electric field and oxygen vacancies in BT for facilitating fast charge separation and promoting N_2_ adsorption and activation under thesynergistic effect of external magnetic field. Su et al. fabricate 10 nm BT nanoparticles (NPs) as excellent piezocatalyst with highly effective longitudinal piezoelectric coefficient, exhibiting the highest hydrogen production efficiency in overall water‐splitting process compared with 200 nm BT nanocubes and BT nanowires.^[^ ^29^ ^]^ Combination of atomic‐resolution high angle annular dark field scanning transmission electron microscopy(HAADF‐STEM), scanning probe microscopy(SPM) and Landau free energy modeling comprehensively reveal that 10 nm BT NPs possess high piezoelectricity originating from a coexistence of multiple phases with low energy barriers and polarization anisotropy, and polarization anisotropy for facilitating polarization rotation, thus boosting the overall water splitting. Despite being considered as excellent photocatalytic and piezocatalytic candidates, few works have been reported on photo‐piezocatalytic plasmonic nanometals/ferroelectrics. Especially, in‐depth mechanistic understandings of the influence of structure optimization and polarization regulation on plasmon‐induced interfacial charge transfer for promoting photo‐piezocatalytic activity are still unclear and vacant.

**Figure 1:**
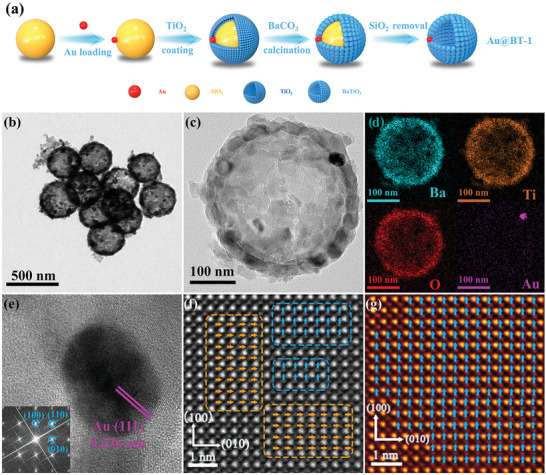
a) Schematic illustration of the fabrication of Au@BT‐1. b,c) TEM image of multiple Au@BT‐1‐P and single Au@BT‐1‐P. d) EDX maps of Ba, Ti, O, and Au from single Au@BT‐1‐P. e) HRTEM image of Au in Au@BT‐1‐P and SAED pattern (inset) of Au@BT‐1‐P. f,g) Atomic‐resolution HAADF‐STEM images of Au@BT‐1 and Au@BT‐1‐P.

In this work, we present an electrostatic adsorption and template method to successfully synthesize a series of yolk‐shell nanostructured catalysts (Au@BT) on the basis of one Au NP to one BT‐HNS (**Figure** **1**a; Figure S1, Supporting Information). Benefiting from abundant interfacial interaction, superior piezoelectric response and augmented polarized electric field, Au NP encapsulated in ferroelectric BT‐HNS (Au@BT‐1) exhibits remarkable photocatalytic, piezocatalytic and coupling visible‐light‐piezocatalytic performance for CO_2_ conversion into CO, which is more active and stable than that of Au NP inside the BT‐HNS (Au@BT‐2) and Au NP separately‐loaded BT‐HNS surface (Au@BT‐3). More importantly, the photo‐piezocatalytic performance of Au@BT‐1 can be further improved by corona poling (Au@BT‐1‐P) with the highest CO production rate of 31.29 µmol g^−1^ h^−1^ without using co‐catalysts or any sacrificial agent under visible light irradiation and ultrasonic vibration. Moreover, Au@BT‐1‐P exhibits outstanding stability of structure and polarization after long cycling. Experimental and theoretical results reveal that the optimized yolk‐in‐shell nanostructure efficiently strengthens the interface interaction, charge transfer and separation, and then enhances CO_2_ adsorption and accelerates the conversion of various intermediates. This work offers an integrated strategy to govern charge migration behaviors based on the rational design, optimized construction and enhanced polarization.

## 2 Results and Discussion

### 2.1 Catalysts Characterization

The Au@BT series were synthesized using a unique multi‐step template method. Tetrabutyl orthotitanate, BaCO_3_, and SiO_2_ were employed as raw materials and internal silica template, respectively. Detailed experimental procedures are demonstrated in Supporting Information (Figure 1a; Figure S1, Supporting Information). The X‐ray diffraction (XRD) patterns of Au@BT‐1, Au@BT‐2, Au@BT‐3, and Au@BT‐1‐P match well with the diffraction pattern of the tetragonal BT phase, without any impurities (Figure S2, Supporting Information). Since the Au NP content, analyzed by inductively coupled plasma atomic emission spectrometry (ICP‐AES), is low (0.1%), no distinctive XRD peaks corresponding to Au NPs are observed. Scanning electron microscopy (SEM, Figure S3, Supporting Information), transmission electron microscopy (TEM, Figure 1b,c; Figures S4a and S5a, Supporting Information), and high‐angle annular dark field‐STEM (HAADF‐STEM, Figures S4b, S5b, and S6, Supporting Information) images reveal that all the synthesized samples possessed a yolk‐shell nanostructure, featuring single Au NP (diameter of ≈20 nm) encapsulated within one BT‐HNS with an internal diameter of ≈270 nm and an external diameter of ≈350 nm. The only variation among the samples is the location of the Au NPs. Additionally, corona poling is proved as a non‐destructive treatment process. TEM energy dispersive X‐ray (EDX) elemental mapping of the Au@BT series (Figure 1d; Figures S4c and S5c, Supporting Information) clearly demonstrates the distribution of Ba, Ti, O, and Au elements within the selected regions, thereby further confirming the diverse growth and distribution patterns of the Au NPs. High‐resolution TEM images display typical Au (111) and BT (010) crystal planes with interplanar spacings of 0.236 nm and 0.399 nm, respectively (Figure 1e; Figure S7, Supporting Information). This finding is consistent with the selected area electron diffraction (SAED, Figure 1e) pattern obtained from the [001] crystal axis of BT. The N_2_ adsorption‐desorption isotherms of both the pristine Au@BT series and the poled Au@BT series feature a typical type IV isotherm with an H3 hysteresis loop, indicating a mesoporous structure (Figure S8, Supporting Information). Moreover, the negligible difference in specific surface area between the two series further confirmed that the corona poling process is non‐destructive to catalyst structure. Notably, all the yolk‐shell structure samples present specific surface areas of ≈15 m^2^ g^−1^ and average pore sizes of ≈25 nm (Table S1, Supporting Information), which is significantly higher than those of BT prepared by solid‐state reaction (BT‐SSR), highlighting that this unique structure can provide abundant mass transfer path of reactants and surface reactive sites internal and external. To gain a deeper understanding of the effect of corona poling on microstructured domains at the atomic scale, aberration‐corrected scanning transmission electron microscopy (ACTEM) was employed to investigate the local polarization states of Au@BT‐1 and Au@BT‐1‐P. As observed in the HAADF‐STEM image (Figure 1f,g), both Ba and Ti atoms show a uniform arrangement in Au@BT‐1 and Au@BT‐1‐P, and A‐site (Ba) atomic columns are darker than those of B‐site (Ti) as HAADF‐STEM is sensitive to heavy elements. The displacement direction of Ti atoms toward the corners of their four nearest neighboring A‐site cations in the unit cell corresponded to the spontaneous polarization in ferroelectric BT, which is crucial for understanding the local polarization structure.^[^ ^30^ ^]^ In stark contrast to Au@BT‐1, the polarization vectors in Au@BT‐1‐P displayed clear alignment parallel to the [001]‐type polar direction, indicating a monodomain polarization state and thus suggesting a stronger polarization after corona poling.

**Figure 2:**
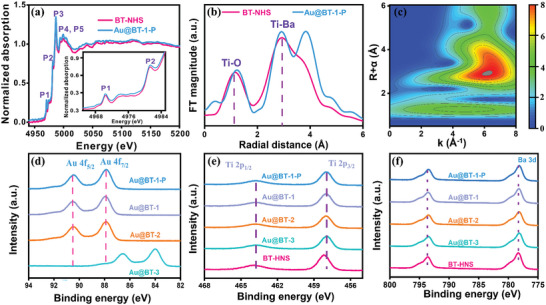
a) Normalized Ti K‐edge XANES spectra and b) k^3^‐weighted Fourier transformed EXAFS of BT‐HNS and Au@BT‐1‐P. c) WT‐EXAFS of Au@BT‐1‐P. XPS spectra of d) Au 4f, e) Ti 2p, and f) Ba 3d of BT‐HNS, Au@BT‐3, Au@BT‐2, Au@BT‐1, and Au@BT‐1‐P.

To investigate the interaction between atoms and the local coordination environment, extended X‐ray absorption fine structure spectroscopy (EXAFS) of the Ti K‐edge in BT‐HNS and Au@BT‐1‐P was analyzed at room temperature. As shown in **Figure** **2**a, the normalized Ti K‐edge XANES spectra of BTO‐HNS and Au@BT‐1‐P both include five typical characteristic absorption peak P1, P2, P3, P4, and P5. Compared with BT‐HNS, the Ti K‐edge of Au@BT‐1‐P slightly shifts to lower energies, implying the change of local atomic coordination environment. Notably, the intensity of pre‐edge peak P1 is directly associated with the displacements of Ti off‐center of oxygen octahedral, and the slight increase of P1 intensity in Au@BT‐1‐P reveals a larger distortion of TiO_6_ octahedral caused by the introduction of Au NPs and corona poling treatment.^[^ ^31^ ^]^ The presentation of peak 2 and peak 3 is caused by electric dipole‐allowed transition of 1s core electrons to an unoccupied 4p bound state.^[^ ^31^ ^]^ Peak 4 and peak 5 as post‐edge characteristic peaks indicate that the Ti ions in BT‐HNS and Au@BT‐1‐P were in Ti^4+^ valence state.^[^ ^32^ ^]^ Further analysis of the Fourier transformed (FT) data and EXAFS wavelet transforms (WT) revealed that the radial distance of Ti‐O in Au@BT‐1‐P is larger than that in BT‐HNS, indicating intimate interface interactions between Au and O atoms (Figure 2b,c; Figure S9, Supporting Information).

X‐ray photoelectron spectroscopy (XPS) was used to analyze the surface composition and chemical states of the elements in Au@BT‐1, Au@BT‐2, Au@BT‐3, and Au@BT‐1‐P. As it can be seen in Figure S10 (Supporting Information), Ba 3d, Ti 2p, Au 4f and O 1s can be found in the survey XPS spectra for all samples. For Au@BT‐2, Au@BT‐1, and Au@BT‐1‐P, the peaks centered at 87.9 and 90.4 eV belong to the Au^3+^ 4f_7/2_ and Au 4f_5/2_, showing an evident shift to higher binding energies compared to Au0 NPs in Au@BT‐3. This shift suggests electron transfer from Au NPs to adjacent O atoms, indicating an interaction between Au and O (Figure 2d).^[^ ^33^ ^]^ Similarly, the shift in the binding energies of Ba and Ti atoms in Figure 2e,f demonstrates the change in the number of coordinating oxygen atoms. O 1s peaks present more negative shift in Au@BT‐2, Au@BT‐1, and Au@BT‐1‐P than Au@BT‐3, implying stronger interface interactions and greater electron transfer from Au NPs in these samples (Figure S11, Supporting Information). It has been reported that octahedral distortion implies the development of oxygen vacancies.^[^ ^34^ ^]^ As shown in Figure S12 (Supporting Information), the evident oxygen vacancy signals are observed in EPR spectra for BT‐HNS, Au@BT‐1, and Au@BT‐1‐P.However, the signals of oxygen vacancies in Au@BT‐1 and Au@BT‐1‐P are weaker compared to BT‐HNS, indicating that the presence of Au NPs effectively reduces the concentration of oxygen vacancies. Notably, Au@BT‐1‐P exhibits slightly enhanced signal strength compared to Au@BT‐1, resulting from stronger octahedral distortion after corona poling treatment.

### 2.2 Enhanced Ferroelectric Polarization Promoting the Charge Separation

**Figure 3:**
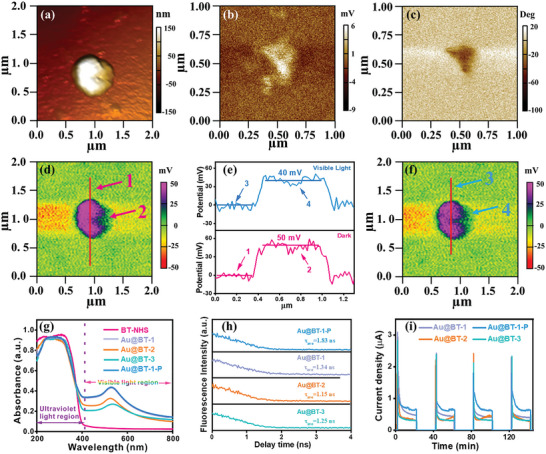
a) AFM image of Au@BT‐1‐P. The PFM b) amplitude and c) phase mapping of Au@BT‐1‐P. d) Surface potential of Au@BT‐1‐P in dark, f) under visible light, and e) corresponding charge difference profile. g) UV–vis diffuse reflectance spectra (DRS) of BT‐HNS, Au@BT‐1, Au@BT‐2, Au@BT‐3, and Au@BT‐1‐P. h) Time‐resolved photoluminescence curves and i) photo‐piezo‐current responses of Au@BT‐1, Au@BT‐2, Au@BT‐3, and Au@BT‐1‐P under ultrasonic vibration and visible light irradiation.

To get in‐depth cognition on the effect of ferroelectric spontaneous polarization in charge behavior, piezoresponse force microscope (PFM) and Kelvin probe force microscopy (KPFM) were used to study the ferroelectric properties of Au@BT‐1‐P. The topography image clearly depicts spherical particles, remaining consistent with the SEM and TEM characterizations (**Figure** **3**a). PFM amplitude mapping and phase mapping display obvious regional color contrast, indicating the different polarization orientations and favorable piezoelectric response in Au@BT‐1‐P (Figure 3b,c). The typical butterfly shape amplitude curve and standard hysteresis *P*–*E* loop with 180° phase switch further reveal the excellent ferroelectric and piezoelectric properties on Au@BT‐1‐P (Figure S13, Supporting Information). The surface charge images provide valuable insights into the charge distributions on the surfaces of BT‐HNS and Au@BT‐1‐P. Both BT‐HNS and Au@BT‐1‐P exhibit different charge distributions, indicating the presence of polar surfaces with a significant plenty of space charges on the ferroelectrics under dark.^[^ ^35^ ^]^ Notably, the corona poling treatment of Au@BT‐1‐P leads to a higher surface potential than BT‐HNS, suggesting the generation of a stronger built‐in electric field (Figure 3d,e; Figure S14a,b, Supporting Information). Interestingly, BT‐HNS shows negligible changes in surface charge under visible light irradiation (Figure S14b,c, Supporting Information). In contrast, Au@BT‐1‐P shows a ≈10 mV decreases at the region of positive potentials (Figure 3e,f). This resulting from the fast migration of visible‐light‐excited electrons from Au NP to BTO‐HNS facilitated by enhanced polarization electric field and efficient plasmon‐induced interfacial charge transfer pathway.

The UV/vis diffuse reflectance spectra (DRS) analysis reveals important information about the optical properties of the materials (Figure 3g). The absorption edge of BT‐HNS is observed at ≈400 nm in the near‐UV region, indicating a bandgap energy of 3.02 eV (Figure S15a, Supporting Information).^[^ ^36^ ^]^ Particularly, a new broad absorption band in the visible light region observed at ≈550 nm can be attributed to the LSPR absorption of the Au NPs in Au@BT series, strongly confirming that the photo‐response range is extended to visible light by introducing Au NPs. In addition, obvious UV shifts with the location changes of Au NPs is related to the interaction between Au NPs and BTO‐HNS. Mott–Schottky plots are employed to determine the flat band potential of BT‐HNS as −0.69 eV versus SCE (Figure S15b, Supporting Information), indicating that BT‐HNS has a sufficient negative conduction band (CB) position for CO_2_ reduction.

Meanwhile, the time‐resolved fluorescence decay spectroscopy, open‐circuit potential, and electrochemical test are conducted to systematically investigate the synergistic effect of the unique yolk‐in‐shell nanostructure and enhanced piezoelectric polarization in significantly promoting the separation and transportation of plasmon‐induced interfacial charges. Time‐resolved fluorescence decay spectroscopy (Figure 3h) revealed that Au@BT‐1 exhibits an average lifetime of 1.34 ns, longer than that of Au@BT‐2 (1.15 ns) and Au@BT‐3 (1.25 ns). Corona poling treatment further prolongs the average lifetime to 1.83 ns for Au@BT‐1‐P, indicating that optimized yolk‐in‐shell nanostructure and enhanced ferroelectric polarization electric field coupled facilitating efficient separation and migration of photogenerated carriers. Photocurrent density shows that Au@BT‐1‐P owns the highest current density, manifesting the highest charge separation efficiency (Figure S16, Supporting Information). Open‐circuit potential was employed to inspect the photoinduced charge carriers transfer behavior between Au NPs and BT‐HNS. The curves of open‐circuit potential of BT‐HNS, Au@BT‐1, and Au@BT‐1‐P under the monochromatic light of 550 nm are shown in Figure S17 (Supporting Information). Clearly, Au@BT‐1‐P presents the highest output photovoltage, demonstrating that the enhanced built‐in electric field accelerates plasmon‐induced electrons transfer from Au NPs to BT‐HNS, leading to increased output photovoltage. Notably, BT‐HNS shows a stronger immediate piezo‐current response than BT‐SSR upon initiating ultrasonic vibration, suggesting BT‐HNS has superior sensitivity to external stress, which generates larger piezoelectric potential to promote the efficient transfer and separation of charges (Figure S18, Supporting Information). Compared to BT‐HNS, Au@BT‐1, Au@BT‐2, and Au@BT‐3, Au@BT‐1 Au@BT‐1‐P also displays the highest piezo‐current response upon initiating ultrasonic vibration (Figure S19, Supporting Information), emphasizing again the critical role of optimizing structural design, besides, enhanced ferroelectric polarization would induce a powerful piezoelectric polarization electric field. More importantly, Au@BT‐1‐P exhibits a significant increase in current density and smallest interfacial resistance under visible light irradiation and ultrasonic vibration (Figure 3i; Figure S20, Supporting Information), suggesting the potential for synergistic effect of photocatalysis and piezocatalysis in Au@BT‐1‐P to achieve an excellent catalytic performance. These findings highlight optimized functional structural design that introduces superior mechanical force‐sensitive response and provides ample interfacial interaction for effective charge transfer. In addition, enhanced ferroelectric polarization can generate stronger polarization under mechanical stress, further promoting efficient separation of charge carriers.

### 2.3 CO_2_ Reduction Performance

**Figure 4:**
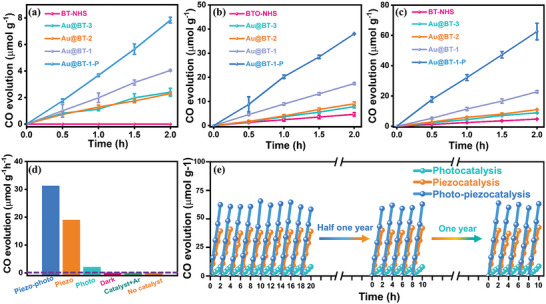
a) Photocatalytic, b) piezocatalytic, and c) photo‐piezocatalytic CO production curves over BT‐NHS, Au@BT‐3, Au@BT‐2, Au@BT‐1, and Au@BT‐1‐P under visible light irradiation, ultrasonic vibration, and ultrasonic vibration and visible light irradiation. d) The corresponding rates over Au@BT‐1‐P under different conditions. e) The cycling tests of CO_2_ reduction into CO over Au@BT‐1‐P.

To underscore the advantage of the optimized structural design and effectual charge‐transport behavior for photocatalytic reaction, the CO_2_ reduction activity of the synthesized samples was evaluated in pure aqueous solution under individual visible light irradiation and ultrasonic vibration, as well as simultaneous visible light irradiation and ultrasonic vibration, without using sacrificial agents. Remarkably, all the Au@BT series samples exhibit CO_2_ reduction activity, generating CO and H_2_ as the main products, with no detectable liquid products (Figure S21, Supporting Information). Among the samples, Au@BT‐1 demonstrates superior photocatalytic CO_2_ reduction performance, with a CO evolution rate of 2.02 µmol g^−1^ h^−1^. This rate is ≈1.75 times and 1.68 times higher than that of Au@BT‐2 (1.15 µmol g^−1^ h^−1^) and Au@BT‐3 (1.20 µmol g^−1^ h^−1^), respectively (**Figure**  **4**a; Figure S22a and Table S3, Supporting Information). Moreover, Au@BT‐1 also delivers the highest photocatalytic H_2_ yield of 1.65 µmol g^−1^ h^−1^, better than that of Au@BT‐2 (1.09 µmol g^−1^ h^−1^) and Au@BT‐3 (1.21 µmol g^−1^ h^−1^) in Figure S23a and Table S4 (Supporting Information). These results suggest that the optimized nanostructure of Au@BT‐1 enables enhanced visible light absorption and efficient plasmon‐induced interfacial charge‐transfer pathways. In contrast, BT‐HNS does not exhibit any detectable CO, H_2_ and other detectable products due to the lack of visible light response. Notably, CO and H_2_ yields gain further promotion for Au@BT‐1‐P (3.92 and 2.59 µmol g^−1^ h^−1^) Au@BT‐2‐P (2.20 and 2.08 µmol g^−1^ h^−1^) and Au@BT‐3‐P (2.15 and 1.90 µmol g^−1^ h^−1^) after corona poling. Among them, Au@BT‐1‐P exhibits the highest CO evolution rate, which is approximately two times that of Au@BT‐1 (Figure 4a; Figures S22a and S23a, Supporting Information), further revealing significant role of reinforced built‐in electric field in boosting photocatalytic CO_2_ reduction activity. Meanwhile, the significant CO evolution rate increase of Au@BT‐1‐P is 3.92 mmol g_Au‐cat_^−1^ h^−1^ based on the content of Au NPs, exceeding most reported state‐of‐the‐art plasmon‐mediated photocatalysts (Figure S24, Supporting Information). The apparent quantum efficiency (AQE) with 550 nm monochromatic irradiation for Au@BT‐1‐P is determined to be ≈0.12%.

Similarly, CO and H_2_ are still the main detectable products for all as‐synthesized samples when subjected to ultrasonic vibration. CO production rates of BT‐HNS is determined at 2.33 µmol g^−1^ h^−1^, which is ≈16 times higher than that of BT‐SSR (0.15 µmol g^−1^ h^−1^), highlighting again that the hollow nanosphere shell possesses excellent sensitivity to external stress with improved mechanical energy collection efficiency (Figures S22b and S23b, Supporting Information). In addition, Au@BT‐1, Au@BT‐2, and Au@BT‐3 present enhanced piezocatalytic activity compared to that of BT‐HNS. Au@BT‐1 shows the highest piezocatalytic CO_2_ activity with the CO and H_2_ yield rate of 8.71 and 6.45 µmol g^−1^ h^−1^, underlining the important role of introducing Au NPs in facilitating piezoelectric catalytic activity. Furthermore, corona poling of Au@BT‐1‐P. Au@BT‐2‐P, and Au@BT‐3‐P finally result in significant boost for CO and H_2_ evolution rates. Particularly, Au@BT‐1‐P owns the highest CO and H_2_ evolution rate of 19.02 and 11.38 µmol g^−1^ h^−1^ (Figure 4b; Figures S22b and S23b, Supporting Information). To further investigate the photo‐piezocatalytic performance of all samples, CO_2_ reduction experiments are carried out under visible light irradiation and ultrasonic vibration. As illustrated in Figure 4c and Figures S22c and S23c (Supporting Information), all the Au@BT series samples present enhanced photo‐piezocatalytic performance in CO_2_ reduction. Remarkably, the synergistically optimized CO and H_2_ production rates of 31.29 and 19.38 µmol g^−1^ h^−1^ are determined for Au@BT‐1‐P, much higher than other samples. Additionally, such prominent performance of CO yield for Au@BT‐1‐P are ≈223.5 times, 10.6 times, and 2.8 times higher than that of BT‐SSR, BT‐NHS, and Au@BT‐1, respectively, perfectly corresponding to the determining factor of the rational structural design, optimized polarization regulation strategy, and resultful charge carrier dynamics modulation in photo‐piezocatalytic CO_2_ reduction activity.

In order to exclude the influence of potential intra system contamination in the preparation, cleaning and catalytic process of catalysts, control experiments and the ^13^C isotopic detecting tests were conducted to confirm the carbon source. As illustrated in Figure 4d, no CO and any other products are detected under Ar atmosphere instead of CO_2_ pre‐purging, in the dark or without catalysts, indicating that the interferential carbon contaminants are not introduced during the whole experiment. When labeled carbon dioxide (^13^CO_2_, *m*/*z* = 45) is purged into the reactor as the carbon source to trace the conversion product, ^13^CO (*m*/*z* = 29) is the only reductive product detected by the mass spectroscopy, further demonstrating that evolved product indeed originates from the photo‐piezocatalytic CO_2_ reduction process (Figure S25, Supporting Information). More importantly, Au@BT‐1‐P shows excellent photocatalytic, piezocatalytic and photo‐piezocatalytic stability with slight activity decay after ten successive cycles. Even after half one year and one year, it still maintains highly stable photo‐piezocatalytic activity in twice five cycles (Figure 4e), revealing Au@BT‐1‐P possesses outstanding stability of structure and polarization. Additionally, XRD, XPS, UV–vis diffuse reflectance and Raman spectroscopy before and after 20 cycles display no obvious change, which further demonstrates that the high stability of Au@BT‐1‐P with optimized yolk‐in‐shell nanostructure and enhanced polarization electric field (Figures S26–S29, Supporting Information). Instead, Au@BT‐2‐P and Au@BT‐3‐P present apparent decrease in CO_2_ reduction under different conditions due to the faint electrostatics action and damaged yolk‐shell structure after three cycles (Figure S30, Supporting Information). In addition, CO_2_ temperature programmed desorption (TPD‐CO_2_) test was carried out to investigate the surface basic sites and the interaction of CO_2_ on all samples. Generally, there are three types of desorption peaks assigned to weak (<200°C), moderate (200—400 °C), and strong (>400°C) basic sites, respectively.^[^ ^37^ ^]^ In comparison with BT‐HNS and Au@BT‐1, BTO‐HNS‐P and Au@BT‐1‐P display stronger chemisorption of CO_2_ at moderate and strong basic sites, and desorption peaks show an obvious shift to higher temperature, indicating that corona poling process can greatly advance the CO_2_ chemical adsorption capacities and effectively promote the continuous transformation into intermediate species (Figure S31, Supporting Information).

### 2.4 COMSOL Simulation of Polarization Electric Field and Catalytic Mechanism by DFT Calculations

**Figure 5:**
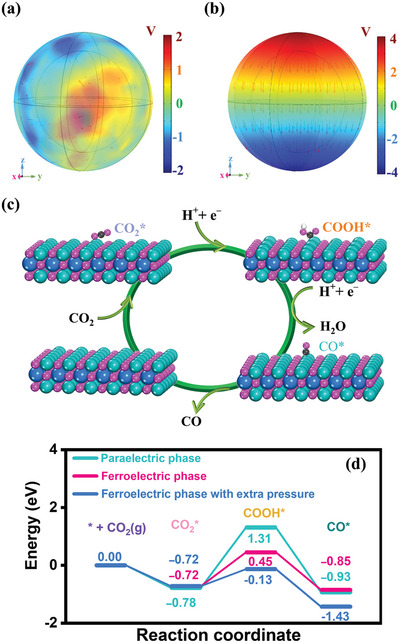
COMSOL simulation of polarized electric field on BT hollow: a) un‐poled, b) fully poled. DFT calculations of BT. c) Optimized structures of CO_2_ reduction to CO on the surface of BT. Color code: Ba‐blue, Ti‐dark cyan, O‐magenta, C‐black, H‐white. d) Free energy diagram of CO_2_ reduction to CO on paraelectric, ferroelectric BT, and ferroelectric BT with extra pressure.

The distribution difference of the electric field intensity resulting from spontaneous polarization in the initial and poled BT hollow nanosphere is simulated by the finite element method (FEM) using COMSOL software (**Figure** **5**a,b). The simulated geometrical size of the BT hollow nanosphere is based on TEM results, with dimensions of 270 nm inside and 350 nm outside. In the original BT hollow nanosphere, the domain orientations are disordered, leading to a chaotic internal electric field. Obviously, the domains switched to align and maintained a highly uniform configuration under the influence of the poling voltage, which creates a strong potential difference of 8 V served as an effective driving force for enhancing the separation of photogenerated charge carriers during the catalytic process. To better understand the crucial role of ferroelectric spontaneous polarization in enhancing the adsorption and activation of CO_2_ molecules, we conducted Density Functional Theory (DFT) calculations to simulate the key reaction processes occurring at the catalyst interface. Figure 5c,d illustrates the calculated Gibbs free energy profiles and corresponding intermediate configurations for the paraelectric Au@BT, ferroelectric Au@BT, and ferroelectric Au@BT with extra pressure, respectively. The results clearly demonstrate that the rate‐determining step, involving the hydrogenation of adsorbed CO_2_ to form *COOH, exhibits a significantly lower free energy barrier in ferroelectric BT (1.17 eV) compared to paraelectric Au@BT (2.09 eV). Especially, the free energy barrier drastically decreases (0.59 eV) when applying a simulated pressure on ferroelectric BT, suggesting ultrasonic vibration induced piezoelectric polarization can effectively activate key intermediates to boost effortless CO_2_ reductions to CO. This observation also aligns with CO_2_ Temperature Programmed Desorption (CO_2_‐TPD) experiments, further indicating the higher activity facilitated by the establishment and enhancement of polarization‐induced electric field in the CO_2_ reduction process. Furthermore, to gain deeper insights into the apparent charge transfer between Au nanoparticles (Au NPs) and BT, we calculated the charge density distributions of the conduction band edge within a 0.2 eV energy window relative to the valence band maximum for BT and Au@BT, as shown in Figures S32 and S33 (Supporting Information), respectively. The comparison reveals that in BT, the conduction band density primarily resides in the inner layers of Ti (Figure S32a, Supporting Information), whereas in the presence of Au, the exposed Ti layer of Au@BT exhibits a considerable distribution of conduction band states (Figure S33a, Supporting Information). This comparison indicates that the interfacial interaction between Au NPs and BT creates more available states near the conduction band edge of BT, potentially enhancing the generation of photogenerated electrons. The aforementioned theoretical results collectively highlight the significance of enhanced induced polarization electric field and intimate interface contacts in the efficient adsorption and activation of CO_2_, ultimately leading to a significant enhancement in catalytic performance.

## 3 Conclusion

In summary, a series of yolk‐shell nanostructured catalysts (Au@BT), each consisting of a single Au NP and one ferroelectric BT‐HNS were synthesized by template method. These catalysts are highly efficient and super stable for coupling visible light and mechanical energy to drive CO_2_ conversion. Among this series of catalysts, Au@BT‐1‐P, with its precisely designed yolk‐in‐shell nanostructure, integrates abundant interfacial interaction and an enhanced polarized electric field. It presents the highest CO production rate of 31.29 µmol g^−1^ h^−1^ without using any sacrificial agent. The experimental and theoretical results reveal that outstanding functional structural design and reinforced polarization electric field can create excellent mechanical force sensitive response and ultra‐high structural stability, thereby providing abundant plasmon‐induced interfacial charge‐transfer pathway and accelerating the transfer of photogenerated electrons from Au NPs to BT‐HNS. More importantly, the enhancement of ferroelectric polarization electric field not only serves as powerful driving force promoting carrier migration, but also lowers the energy barrier of forming the key intermediate COOH* in CO_2_ reduction reaction. This study pioneers the potential and deep‐level cognition of optimized structural design and polarized electric field regulation for developing high‐performance and multi‐responsive catalysts for green energy conversion.

## Conflict of Interest

The authors declare no conflict of interest.

## Supporting information



Supporting Information

## Data Availability

The data that support the findings of this study are available from the corresponding author upon reasonable request.

## References

[1] Z. Jiang , X. Xu , Y. Ma , H. S. Cho , D. Ding , C. Wang , J. Wu , P. Oleynikov , M. Jia , J. Cheng , Nature 2020, 586, 549. 32906144 10.1038/s41586-020-2738-2

[2] J. Huang , F. Li , A. Ozden , A. Sedighian Rasouli , F. García de Arquer , S. Liu , S. Zhang , M. Luo , X. Wang , Y. Lum , Science 2021, 372, 1074. 34083485 10.1126/science.abg6582

[3] S. Zhang , R. Chen , D. Kong , Y. Chen , W. Liu , D. Jiang , W. Zhao , C. Chang , Y. Yang , Y. Liu , D. Wei , Nat. Nanotech. 2024, 19, 1323. 10.1038/s41565-024-01707-0 38965348

[4] H. Ni , Y. Yuan , M. Li , Y. Zhu , X. Ge , J. Yin , C. P. Dessai , L. Wang , J. Cheng , Nat. Photonics 2024,18, 944.

[5] Y. Yuan , G. Yan , C. Dreessen , T. Rudolph , M. Hülsbeck , B. Klingebiel , J. Ye , U. Rau , T. Kirchartz , Nat. Mater. 2024, 23, 391. 38195863 10.1038/s41563-023-01771-2 PMC10917677

[6] B. Lee , E. Gong , M. Kim , S. Park , H. Kim , J. Lee , E. Jung , C. Lee , J. Bok , Y. Jung , Energy Environ. Sci. 2022, 15, 601.

[7] E. Gong , S. Ali , C. Hiragond , H. Kim , N. Powar , D. Kim , H. Kim , S. In , Energy Environ. Sci. 2022, 15, 880.

[8] H. Yu , F. Chen , X. Li , H. Huang , Q. Zhang , S. Su , K. Wang , E. Mao , B. Mei , G. Mul , Nat. Commun. 2021, 12, 1. 34321482 10.1038/s41467-021-24882-3 PMC8319429

[9] P. Zhang , T. Wang , J. Gong , Adv. Mater. 2015, 27, 5328. 26265309 10.1002/adma.201500888

[10] U. Aslam , V. G. Rao , S. Chavez , S. Linic , Nat. Catal. 2018, 1, 656.

[11] W. Shangguan , Q. Liu , Y. Wang , N. Sun , Y. Liu , R. Zhao , Y. Li , C. Wang , J. Zhao , Nat. Commun. 2022, 13, 1. 35794088 10.1038/s41467-022-31474-2 PMC9259601

[12] X. Meng , L. Liu , S. Ouyang , H. Xu , D. Wang , N. Zhao , J. Ye , Adv. Mater. 2016, 28, 6781. 27185493 10.1002/adma.201600305

[13] A. Furube , L. Du , K. Hara , R. Katoh , M. Tachiya , J. Am. Chem. Soc. 2007, 129, 14852. 17994750 10.1021/ja076134v

[14] C. Clavero , Nat. Photonics 2014, 8, 95.

[15] C. Guo , C. Li , Adv. Funct. Mater. 2016, 26, 8824. 10.1002/adfm.201504185 PMC5077153 27790080

[16] L. Lin , J. Song , H. Yang , X. Chen , Adv. Mater. 2018, 30, 1704639 10.1002/adma.201704639 29280201

[17] S. Das , J. Pérez‐Ramírez , J. Gong , N. Dewangan , K. Hidajat , B. Gates , S. Kawi , Chem. Soc. Rev. 2020, 49, 2937. 32407432 10.1039/c9cs00713j

[18] J. Han , M. Wang , R. Chen , N. Han , R. Guo , Chem. Commun. 2014, 50, 8295. 10.1039/c4cc01532k 24777116

[19] X. Sun , J. Han , R. Guo , Front. Chem. 2020, 8, 606044. 33330401 10.3389/fchem.2020.606044 PMC7734176

[20] J. Hu , R. Li , J. Han , J. Sun , Y. Wang , L. Yu , R. Guo , J. Mater. Chem. A. 2020, 8, 10217.

[21] J. Hu , R. Zhao , H. Li , Z. Xu , H. Dai , H. Gao , H. Yu , Z. Wang , Y. Wang , Y. Liu , J. Han , R. Guo , Appl. Catal. B: Environ. 2022, 303, 120869.

[22] F. Chen , H. Huang , L. Guo , Y. Zhang , T. Ma , Angew. Chem., Int. Ed. 2019, 58, 10061. 10.1002/anie.201901361 30794343

[23] J. Jing , J. Yang , W. Li , Z. Wu , Y. Zhu , Adv. Mater. 2022, 34, 2106807. 10.1002/adma.202106807 34738259

[24] L. Pan , S. Sun , Y. Chen , P. Wang , J. Wang , X. Zhang , J. Zou , Z. L. Wang , Adv. Energy Mater. 2020, 10, 2000214.

[25] H. Li , Y. Sang , S. Chang , X. Huang , Y. Zhang , R. Yang , H. Jiang , H. Liu , Z. Wang , Nano Lett. 2015, 15, 2372. 25803813 10.1021/nl504630j

[26] M. Mirseraji , M. G. Shahraki , Physica B. 2018, 538, 120.

[27] Q. Zhang , Y. Jia , W. Wu , C. Pei , G. Zhu , Z. Wu , L. Zhang , W. Fan , Z. Wu , Nano Energy 2023, 113, 108507.

[28] Z. Zhao , D. Wang , R. Gao , G. Wen , M. Feng , G. Song , J. Zhu , D. Luo , H. Tan , X. Ge , W. Zhang , Y. Zhang , L. Zheng , H. Li , Z. Chen , Angew. Chem., Int. Ed. 2021, 60, 12144. 10.1002/anie.202100726 33605019

[29] R. Su , H. A. Hsain , M. Wu , D. Zhang , X. Hu , Z. Wang , X. Wang , F. Li , X. Chen , L. Zhu , Y. Yang , Y. Yang , X. Lou , S. Pennycook , Angew. Chem., Int. Ed. 2019, 58, 15076. 10.1002/anie.201907695 31404487

[30] M. Polking , M. Han , A. Yourdkhani , V. Petkov , C. Kisielowski , V. Volkov , Y. Zhu , G. Caruntu , A. Paul Alivisatos , R. Ramesh , Nat. Mater. 2012, 11, 700. 22772655 10.1038/nmat3371

[31] P. Phaktapha , J. Jutimoosik , A. Bootchanont , P. Kidkhunthod , S. Rujirawat , R. Yimnirun , Integr. Ferroelectr. 2017, 177, 74.

[32] J. Padchasri , N. Triamnak , T. Sareein , J. Jutimoosik , S. Tongsaeng , A. Bootchanont , P. Kidkhunthod , S. Rujirawat , P. Manyum , R. Yimnirun , Radiat. Phys. Chem. 2021, 188, 109657.

[33] H. Qian , F. Meng , C. Yang , X. Yan , Angew. Chem., Int. Ed. 2020, 59, 17607. 10.1002/anie.202006535 32497359

[34] S. Köhne , O. Schirmer , H. Hesse , T. W. Kool , V. Vikhnin , J. Supercond. 1999, 12, 193.

[35] R. Su , Z. Wang , L. Zhu , Y. Pan , D. Zhang , H. Wen , Z. D. Luo , L. Li , F.t. Li , M. Wu , Angew. Chem., Int. Ed. 2021, 60, 16019. 10.1002/anie.202103112 33871146

[36] B. Kwak , M. Kang , J. Nanosci. Nanotechnol. 2017, 17, 7351.

[37] G. Siakavelas , N. Charisiou , S. AlKhoori , A. AlKhoori , V. Sebastian , S. Hinder , M. Baker , I. Yentekakis , K. Polychronopoulou , M. A. Goula , Appl. Catal. B: Environ. 2021, 282, 119562.

