# Supplementary material for: Enhanced Ferroelectric Polarization in Au@BaTiO3 Yolk‐in‐Shell Nanostructure for Synergistic Boosting Visible‐Light‐ Piezocatalytic CO2 Reduction
Source: Adv Sci (Weinh). 2024 Oct 16;11(45):2410357. doi: 10.1002/advs.202410357 (PMC11615802; doi:10.1002/advs.202410357)
Supplement: Supplementary file 1 — Supporting Information [file ADVS-11-2410357-s001.docx]

Supporting Information

**Enhanced ferroelectric polarization in Au@ BaTiO_3_ Yolk-in-Shell Nanostructure for Synergistic Boosting Visible-Light- Piezocatalytic CO_2_ Reduction**

*Jun Hu, Rufang Zhao,* *Jingren Ni, Wei Luo, Hongjian Yu*,* *Hongwei Huang*, Boyuan Wu, Yang Wang*, Jie Han***, Rong Guo*

J. Hu, J. R. Ni, Dr. H. J. Yu, Prof. Y. Wang, Prof. J. Han, Prof. R. Guo

School of Chemistry and Chemical Engineering, Yangzhou University, Yangzhou 225002, China

E-mail: yhj@yzu.edu.cn; yangwang@yzu.edu.cn; hanjie@yzu.edu.cn

Dr. R. F. Zhao

Hubei Key Laboratory of Pollutant Analysis & Reuse Technology, College of Chemistry and Chemical Engineering, Hubei Normal University, Huangshi 435002, Hubei, China

Dr. W. Luo

Hubei Key Laboratory of Hydropower Engineering Construction and Management, and College of Hydraulic & Environmental Engineering, China Three Gorges University, Yichang 443002, Hubei, China

Prof. H. W. Huang

Engineering Research Center of Ministry of Education for Geological Carbon Storage and Low Carbon Utilization of Resources, Beijing Key Laboratory of Materials Utilization of Nonmetallic Minerals and Solid Wastes, National Laboratory of Mineral Materials, School of Material Sciences and Technology, China University of Geosciences (Beijing), Beijing 100083, China

E-mail: hhw@cugb.edu.cn

B.Y. Wu

Department of Physics, The Chinese University of Hong Kong, Shatin, Hong Kong SAR, China

**Table of Contents**

**1. Experimental Section** 5

**1.1 Sample preparation** 5

**1.2 Sample characterization.** 7

**1.3 Photocatalytic, piezocatalytic and photo-piezocatalytic CO_2_ reduction test.** 7

**1.4 Electrochemical tests.** 8

**1.5 COMSOL simulation and density functional theory (DFT) calculations** 9

**2.** **Supplementary Figures** 12

**Figure S1.** Schematic illustration of the fabrication of (a) Au@BT-2 and (b) Au@BT-3. 12

**Figure S2.** XRD patterns of Au@BT-1, Au@BT-2, Au@BT-3, and Au@BT-1-P. 13

**Figure S3.** (a) SEM images of Au@BT-1, (b) Au@BT-2, (c) Au@BT-3, and (d) Au@BT-1-P. 14

**Figure S4.** (a) TEM image, (b) HAADF-STEM image and (c) EDX maps of Ba, Ti, O, and Au from single Au@BT-2. 15

**Figure S5.** (a) TEM image, (b) HAADF-STEM image and (c) EDX maps of Ba, Ti, O, and Au from single Au@BT-3. 16

**Figure S6.** HAADF-STEM image of Au@BT-1-P. 17

**Figure S7.** HRTEM image of BT in Au@BT-1-P. 18

**Figure S8.** (a, b) N_2_ adsorption isotherms and specific surface area of Au@BT-1, Au@BT-2, Au@BT-3, Au@BT-1-P, Au@BT-2-P, and Au@BT-3-P. 19

**Figure S9.** WT-EXAFS of BT-HNS. 20

**Figure S10.** XPS survey spectra of Au@BT-1, Au@BT-2, Au@BT-3, and Au@BT-1-P. 21

**Figure S11.** XPS spectra of O 1s of BT-HNS, Au@BT-1, Au@BT-2, Au@BT-3, and Au@BT-1-P. 22

**Figure S12.** EPR plots of BT-HNS, Au@BT-1 and Au@BT-1-P. 23

**Figure S13.** The butterfly curve and phase hysteresis loop of Au@BT-1-P. 24

**Figure S14.** (a) Surface charge of BT-HNS in dark, (c) under visible light and (b) corresponding charge difference profile. 25

**Figure S15.** (a) Band gap of BT-HNS. (b) Mott-Schottky plots of BT-HNS at frequencies of 100 Hz, 250 Hz and 500 Hz (0.1 M Na_2_SO_4_). 26

**Figure S16.** Photocurrent responses of Au@BT-1, Au@BT-2, Au@BT-3, and Au@BT-1-P under visible light. 27

**Figure S17** Open circle voltage of BT-HNS, Au@BT-1, and Au@BT-1-P under the monochromatic light of 550 nm. 28

**Figure S18.** Piezo-current responses of BT-SSR and BT-NHS under ultrasonic vibration. 29

**Figure S19.** Piezo-current responses of Au@BT-1, Au@BT-2, Au@BT-3 and Au@BT-1-P under ultrasonic vibration. 30

**Figure S20.** EIS Nyquist plots of Au@BT-1, Au@BT-2, Au@BT-3 and Au@BT-1-P under ultrasonic vibration and visible light irradiation. 31

**Figure S21.** ^1^H NMR spectrum of the solution after the catalytic reaction in DMSO. 32

**Figure S22.** (a) Photocatalytic, (b) piezocatalytic and (c) photo-piezocatalytic CO rates over BNT-HNS, Au@BT-2, Au@BT-3, Au@BT-1, Au@BT-2-P, Au@BT-3-P and Au@BT-1-P. 33

**Figure S23.** (a) Photocatalytic, (b) piezocatalytic and (c) photo-piezocatalytic H_2_ rates over BNT-HNS, Au@BT-2, Au@BT-3, Au@BT-1, Au@BT-2-P, Au@BT-3-P, and Au@BT-1-P. 34

**Figure S24.** Photocatalytic CO evolution performance comparison of Au@BT-1-P with the catalysts reported in literatures. 35

**Figure S25.** MS spectra for photo-piezocatalytic CO_2_ reduction of Au@BT-1-P with using ^13^CO_2_ as the reacting gas. 36

**Figure S26.** XRD pattern of Au@BT-1-P after 20 cycles. 37

**Figure S27.** XPS spectra of (a) Au 4f, (b) Ba 3d, (c) Ti 2p and (d) O 1s of Au@BT-1-P after 20 cycles. 38

**Figure S28.** UV/Vis diffuse reflectance spectra of Au@BT-1-P after 20 cycles. 39

**Figure S29.** Raman spectra of Au@BT-1-P and Au@BT-1-P-R (Au@BT-1-P after 20 cycles). 40

**Figure S30.** CO production through 3 reaction cycles using (a) Au@BT-2-P and (b) Au@BT-3-P under different reaction conditions. 41

**Figure S31.** CO_2_-TPD profiles of BT-HNS, BT-HNS-P, Au@BT-1, and Au@BT-1-P. 42

**Figure S32.** (a) The DFT-calculated charge density of the conduction band edge for BaTiO_3_. Ba, Ti, and O atoms are shown in blue, yellow, dark cyan and magenta, respectively. (b) The DFT-calculated PDOSs for BaTiO_3_. 43

**Figure S33.** (a) The DFT-calculated charge density of the conduction band edge for Au@BaTiO_3_. Ba, Au, Ti, and O atoms are shown in blue, yellow, dark cyan and magenta, respectively. (b) The DFT-calculated PDOSs for Au@BaTiO_3_. 44

**Figure S34.** Sideview of the supercell of slab model of BaTiO_3_ (001) surface used in the DFT calculations. The Ba, Ti and O atoms are indicated by green, light blue and red spheres, respectively. 45

**Figure S35.** Side views of CO_2_ adsorption configuration on fully poled BaTiO_3_ (001) surface. Barium, titanium, oxygen, carbon, and the oxygen atoms in CO_2_ are shown in green, white, red, gray, and magenta, respectively. 46

**Figure S36.** Side views of CO_2_ adsorption configuration on un-poled BaTiO_3_ (001) surface. The color code for atoms is the same as in Figure S35. 47

**Figure S37.** Side view and top view of adsorption configuration of each reaction pathway on un-poled BaTiO_3_ (001) surface. Color code: Ba-green, Ti-grey, O-red, C-black, H-white, O on reactants: pink. 48

**Figure S38.** Side view and top view of adsorption configuration of each reaction pathway on poled BaTiO_3_ (001) surface. The color code for atoms is the same as in Figure S37. 49

**Figure S39.** Side view and top view of adsorption configuration of each reaction pathway on poled BaTiO_3_ (001) surface with extra pressure. The color code for atoms is the same as in Figure S37. 50

**3. Supplementary Tables** 51

**Table S1.** Specific surface area and pore diameter of different catalysts. 51

**Table S2.** Time resolved PL decay parameter for Au@BT-1, Au@BT-2, Au@BT-3 and Au@BT-1-P. 52

**Table S3.** Photocatalytic, piezocatalytic and photo-piezocatalytic CO rates of BT-SSR, BT-NHS, Au@BT-1, Au@BT-2, Au@BT-3, Au@BT-1-P, Au@BT-2-P, and Au@BT-3-P. 53

**Table S4.** Photocatalytic, piezocatalytic and photo-piezocatalytic H_2_ rates of BT-NHS, Au@BT-1, Au@BT-2, Au@BT-3, Au@BT-1-P, Au@BT-2-P and Au@BT-3-P. 54

**Reference** 55

**1. Experimental Section**

**1.1 Sample preparation**

**1.1.1 Chemicals**

All the reagents are used as received in this work without further purification. Tetrabutyl orthotitanate (TBOT), tetraethyl orthosilicate (TEOS, 98 %), triethanolamine (AR), polyvinypyrrolidone (PVP-10), and (3-aminopropyl) triethoxysilane (APTES, 99 %) are purchased from Sigma-Aldrich. NaOH (≥ 97.0 %, pellets), BaCO_3_ (AR), Ethanol (≥ 99.9 %), chloroauric acid (HAuCl_4_·3H_2_O, 98 %), acetonitrile (≥ 99.9 %) and anhydrous sodium sulfate (99.9 %) and other chemicals (analytical pure) are purchased from Sinopharm Reagent (Shanghai, China).

**1.1.2 Synthesis of Au@SiO_2_ core-shell nanocomposites**

Firstly, chloroauric acid aqueous solution (305 μL, 0.1 mol/L) and trisodium citrate aqueous solution (6 mL, 0.005 mol/L) were added in 30 mL boiling water and reflowed for half an hour. After cooling to room temperature, added PVP-10 (0.235 mL, 12.8 g/L) and stirred the obtained colloidal solution for 12 h. Then, centrifuged the colloidal solution and re-dispersed in 4 mL aqueous solution (denoted as Au colloidal solution) for follow-up use. Subsequently, 0.55 mL Au colloidal solution mixed with 3 mL H_2_O, 0.5 mL ammonia, and 20 mL isopropanol, keeping stirring for 30 min. Then, 0.0625 μL TEOS was added to the mixed solution every hour within 4 h. Finally, the obtained Au@SiO_2_ core-shell nanoparticles were washed with deionized water for 3 times and dried at 60 ℃ for 12 h.

**1.1.3 Synthesis of SiO_2_-NH_2_ nanoparticles**

24 mL TEOS was slowly added into the mixed solution containing 480 mL ethanol, 40 mL H_2_O, and 15 mL ammonia, followed by vigorous stirring for 24 h to obtain SiO_2_ nanoparticles.

Then, SiO_2_ nanoparticles were washed with ethanol aqueous solution for 3 times and dried at 60 ℃ for 12 h. Subsequently, 1.0 g SiO_2_ nanoparticles were dispersed with boiling isopropanol (150 °C), then added 0.4 mL APTES and reflowed for 12 h. Finally, the obtained SiO_2_-NH_2_ nanoparticles were washed with deionized water for 3 times and dried at 60 ℃ for 12 h.

**1.1.4 Synthesis of Au@SiO_2_ Janus nanoparticles**

75 mg SiO_2_-NH_2_ nanoparticles were dispersed in aqueous solution (30 mL), followed by adding Au colloidal solution (0.55 mL). Then, the mixed solution was stirred at room temperature for 2 h. Finally, the obtained Au@SiO_2_ Janus nanoparticles were washed with deionized water for 3 times and dried at 60 ℃ for 12 h.

**1.1.5 Synthesis of Au@BT-1 nanostructure**

80 mg Au@SiO_2_ Janus nanoparticles were dispersed in the mixed solution containing with 25 mL ethanol, 7 mL acetonitrile and 0.2 mL ammonia and stirred for 30 min. Subsequently, 0.5 mL TBOT was added slowly into the mixed solution 3 mL ethanol and 1mL acetonitrile, and stirred for 4 h, then the precursor was centrifuged and washed the above slurry for 3 times by ethanol. The precursor was calcined at 500 ℃ (5 ℃/min) for 2 h to obtain SiO_2_@Au@TiO_2_ hybrids. After that, BaCO_3_ and SiO_2_@Au@TiO_2_ hybrids sintered at 1000 ℃ for 4 h in the muffle furnace to prerared SiO_2_@Au@BaTiO_3_. Finally, the target product was obtained by removing the internal silica template via hydrothermal method with adding sodium hydroxide.

**1.1.6 Synthesis of Au@BT-2 nanostructure**

The synthetic procedures of Au@BT-2 nanostructure is same as those of Au@BT-1 nanostructure, except that Au@SiO_2_ core-shell nanoparticles were used instead of Au@SiO_2_ Janus nanoparticles.

**1.1.7 Synthesis of BT-HNS nanostructure**

The synthetic procedures of BT-HNS nanostructure is same as those of Au@BT-1 nanostructure, with thedifference that 80 mg SiO_2_-NH_2_ was added instead of 80 mg Au@SiO_2_ Janus nanoparticles.

**1.1.8 Synthesis of Au@BT-3 nanostructure**

Based on the obtained BT-HNS nanocatalyst, 0.55 ml of Au colloidal solution was added into BT-HNS modified with amino groups reflowed with APTES and stirred for 2 h. Then, Au@BT-3 nanostructure was washed with deionized water for 3 times.

**1.1.9 Synthesis of BT-SSR**

The raw materials of BaCO_3_ and TiO_2_ were were mixed evenly according to the stoichiometric ratio and calcined at 1000 ℃ for 4 h.

**1.1.10 Corona poling** **sample**

50 mg of prepared Au@BaTiO_3_ series were uniformly dispersed on a negative disk-like copper electrode. Then the steel tip electrode was given a voltage of 20 kV between the two electrodes with a 1 cm distance for 30 min to get polarized powder denoted as Au@ BT-1-P, Au@ BT-2-P and Au@ BT-3-P, respectively.

**1.2 Sample characterization.**

X-ray diffraction patterns were conducted on an X-ray diffractometer (D8 Advance, Bruker AXS). The morphologies and structures of the synthesized products were confirmed by field emission scanning electron microscopy (GeminiSEM 300, ZEISS), transmission electron microscopy (Tecnai-12, Philips) and high-resolution transmission electron microscopy (Tecnai G2 F30 S-Twin TEM, FEI). The specific surface area and pore size were evaluated by BET anal2is instrument (Beishide 3H-2000PS2). The light absorption spectroscopy was recorded using a UV-Vis-NIR spectrophotometer (Cary 5000 Varian, Agilent). The composition was investigated by X-ray photoelectron spectroscopy (ESCALAB 250 spectrometer, Thermo Scientific). The room-temperature X-ray absorption fine structure (XAFS) data over the Ti K-edge of BT-HNS and Au@ BT-1-P were collected at 44A beamline of National Synchrotron Radiation Research Center (NSRRC) Taiwan. The data were collected in fluorescence mode using a Lytle detector while the corresponding reference sample were collected in transmission mode. The catalytic liquid phase product was confirmed by NMR spectrometer (AVANCE 600, Bruker). CO_2_ temperature programmed desorption (TPD-CO_2_) of the samples were obtained by using a multifunction chemisorption analyzer (AutoChemII2920, Micromeritics). Piezoresponse force microscopy module (PFM) in atomic force microscopy (SPA-300HV, SEIKO) was used to study the piezoelectricity of samples. Kelvin probe force microscopy (KPFM) was utilized to investigate the surface potential of samples at dark and visible light irradiation, respectively.

**1.3 Photocatalytic, piezocatalytic and photo-piezocatalytic CO_2_ reduction test.**

**1.3.1 Photocatalytic CO_2_ reduction reaction experiments**

The photocatalytic CO_2_ reduction experiments were tested in a custom-made glass reactor with a quartz glass top. Firstly, 5 mg obtained photocatalyst was dispersed into 10 mL of deionized water and put into quartz reactor, purging with high purity CO_2_ gas for 15 min to exclude other gases. Then, A 300 W Xe lamp as the visible light source (PLS-SXE300+, Beijing Perfect light) with a 420 nm cut-off filter was used to irradiate the mixed solution under photoreaction temperature keeping at 20 ℃. Finally, 1 mL resultant gas and 20 μL of liquid were collected at regular intervals and analyzed by a gas chromatography (A917Plus, PANNA) to analyze gas and liquid products.

**1.3.2 Piezocatalytic CO_2_ Reduction Reaction Experiments.**

Similar to the photocatalytic reaction, quartz reactor with catalyst was placed in a fixed position in the ultrasonic cleaner (KQ-300DE 40 kHz, 240 W, Kunshan Ultrasonic Instruments) to apply ultrasonic vibration. Finally, Finally, 1 mL resultant gas and 20 μL of liquid were collected at regular intervals and analyzed by a gas chromatography (A917Plus, PANNA) to analyze gas and liquid products.

**1.3.3 Photo-piezocatalytic CO_2_ Reduction Reaction Experiments**

Typically, a 300 W Xe lamp as the visible light source (PLS-SXE300+, Beijing Perfect light) with a 420 nm cut-off filter and ultrasonic cleaner (KQ-300DE 40 kHz, 240 W, Kunshan Ultrasonic Instruments) were used simultaneously to apply illumination and ultrasonic vibration to the quartz reactor in the photo-piezocatalytic CO_2_ reduction reaction process. Similarly, the gas and liquid products were analyzed by gas chromatography (A917Plus, PANNA) as photocatalytic and piezocatalytic CO_2_ reduction experiments.

1 atm ^13^CO_2_ (Beijing Gaisi Chemical Gases Company) and 10 mL of water are injected into the reactor to trace the carbon sources. The isotope labeling measurement was analyzed by a mass spectrometry (GSD, 350, OmniStar) at room temperature with He as carrier gas. ^13^CO_2_ (Wuhan Isotope Technology Co., Ltd) was used for the detection of carbon source. 1 atm high purity Ar and 10 mL of water were injected into the reactor to detect the influence of potential intrasystem contamination in the preparation, cleaning and catalytic reaction of photocatalysts under the same experimental conditions. Cyclic experiment and storage process: each catalyst was tested for 2 h, then regenerated at 80 °C for 6 h in a vacuum drying oven. The catalyst after 10 successive cycles was stored in a vacuum dryer for every six months for successive five cycles to analyze the optimized yolk-in-shell nanostructure and enhanced polarization electric field.

**1.3.4 The calculation of apparent quantum efficiency (AQE).**

550 nm monochromatic light was used to test the apparent quantum efficiency (AQE) by the following equation:

$AQE\left( \% \right)=\frac{N_{Solar fuels}}{N_{p}}=\frac{2\times number of CO molecules+2\times number of H_{2} molecules}{The number of incident photos} \times100\%$ (1)

**1.4** **Electrochemical tests.**

Photocurrent response and Mott-Schottky curves of samples are carried out on an electrochemical workstation (CHI-760E, Chenhua Instruments) using a three-electrode system. In this system, a saturated calomel electrode (SCE) and platinum (Pt) wire are utilized as the reference electrode and the counter electrode, respectively. The electrolyte solution is 0.1 M Na_2_SO_4_ solution, and the sample films are coated on indium-tin oxide (ITO) glass substrate (20 mm ×40 mm) as the working electrode. And the measurements are conducted at room temperature with a 300 W xenon lamp as the visible light source (PLS-SXE300+, Beijing Perfect light). There is no applied voltage between the electrodes for photocurrent response. Piezo-current response, photo-piezo-current response, electrochemical impedance spectroscopy (EIS) are tested by the same method as photocurrent response under ultrasonic vibration (KQ-300DE 40 kHz, 240 W, Kunshan Ultrasonic Instruments) and under visible light irradiation (PLS-SXE300+, Beijing Perfect light) and ultrasonic vibration, respectively.

**1.5 COMSOL simulation and density functional theory (DFT) calculations**

**1.5.1 COMSOL simulation of finite element method (FEM).**

The distribution difference of the electric field intensity inducing by the orientation of ferroelectric domains on the initial and poled BT-HNS is simulated using electrostatic physical field in the AC/DC module and FEM in COMSOL Multiphysics software. The simulated geometrical model in accordance with TEM results are registered as BT-HNS (270 nm inside and 350 nm outside). Approximately 230 ferroelectric domains are uniformly set in the BT-HNS geometric model. As for poled BT-HNS, the applied electric field intensity (*E*) is 20 kV/cm, the relative dielectric constant(*ε_r_*) is 1500 and the polarization intensity(*P*) is 16 μC/cm^2^. The ferroelectric domains are randomly oriented in the initial BT-HNS, and the applied electric field intensity (*E*) is 0 kV/cm. All relevant parameters are obtained from previous reported and experimental data of this work in the COMSOL simulationprocess.^[1, 2]^ The space potential induced by domain interactions is defined by the following equation:

$\mathbf{D=}\varepsilon_{0}\boldsymbol{E}+\boldsymbol{P}$ (2)

where *D* is electric displacement, *ε_0_* is the permittivity of vacuum, *E* is electric field strength, *P* is polarization strength.

**1.5.2** **Density functional theory (DFT) calculations.**

All calculations are performed under the framework of the density functional theory (DFT) method in the Vienna ab initio simulation package (VASP, version 5.4.4).^[3]^ The exchange and correlation potential is described by the generalized gradient approximation (GGA) using the Perdew-Burke-Ernzerhof (PBE) functional.^[4]^ The projector augmented wave method^[5]^ is used to describe the electron-ion interactions, and a plane-wave kinetic energy cut-off of 450 eV is chosen for the plane-wave expansion. To accurately describe the electronic structure of the strongly correlated material BaTiO_3_ (001), we employed DFT + *U* approach with a value of *U* = 4.0 eV applied to the 3d electrons of Ti, ^[6]^ which is carefully parameterized in order to well reproduce the experimental DOS-related properties. Accordingly, the density of states (DOS) was calculated using the PBE + *U* approach while the total energy calculations and geometry optimizations were performed using the conventional method with PBE functional.^[7]^ The van der Waals interaction is described by the DFT-D3 method of Grimme.^[8]^ We used a 3 × 3 × 1 Monkhorst−Pack *k*-point mesh to sample the Brillouin zone.^[9]^ During the geometric optimizations, all atoms are allowed to relax except the lowest two layers until the convergence tolerance of energy is less than 1×10^-4^ eV.

The BaTiO_3_ (001) surface was modeled by a periodic slab of (2 × 2) supercell containing 7 atomic layers, as shown in Figure S34 below. In this model, we expose the Ti atom layer to the surface, because previous reported literature demonstrates that Ti atom is the active site of carbon dioxide reduction reaction.^[10]^ The BaTiO_3_ (001) surface model is structured from bottom to top as follows: Ti-O, Ba-O, Ti-O, Ba-O, Ti-O, Ba-O, and Ti-O layer. A vacuum space of ca. 15 Å in the *z* direction is placed in the direction perpendicular to the slab, which is large enough to avoid interactions between neighboring BaTiO_3_ (001) images. The slab was first constructed from the optimized bulk structure of BaTiO_3_, whose optimized lattice parameters (*a* = *b* = 4.007 Å, *c* = 4.019 Å) are in good agreement with the experimental values (*a* = *b* = 4.000 Å, *c* = 4.018 Å).^[11]^ In passing, our calculations confirmed the ferroelectric nature of barium titanate; the polarized bulk structure of BaTiO_3_ is indeed energetically more stable than the unpolarized bulk structure by ca. 0.015 eV per unit cell. Then, the slab structure of BaTiO_3_ (001) (Figure S34) was subsequently optimized, allowing the uppermost three layers of atoms to be fully relaxed while the lowermost four atomic layers were kept frozen. To analyze the effect of external pressure on the catalytic activity of the material, we applied an additional pressure of 2 GPa to the structure along the reaction path. The volume of the simulation cell was kept fixed during the application of this pressure.

For the CO_2_ reduction over BaTiO_3_ (001), we have considered all elementary steps as follows:

CO_2_ + * → CO_2_* (3)

CO_2_* + H^+^ + e^–^ → COOH* (4)

COOH* + H^+^ + e^–^ → CO* + H_2_O (5)

CO* → * + CO (6)

It is important to highlight that, in the quest to simulate the most favorable adsorption configurations and identify potential adsorption sites for CO_2_ on the BaTiO_3_ (001) surface, a comprehensive evaluation of diverse adsorption scenarios was conducted, as illustrated in Figure S35 and S36. Meanwhile, the adsorption points in the subsequent reaction pathway of paraelectric, ferroelectric BT and ferroelectric BT with extra pressure are shown in Figure S37-S39. The findings demonstrate that CO_2_ preferentially adsorbs in an inclined orientation above the titanium (Ti) atoms on the BaTiO_3_ (001) surfaces, whether un-poled or fully poled, facilitating subsequent reaction processes.

**2.** **Supplementary Figures**


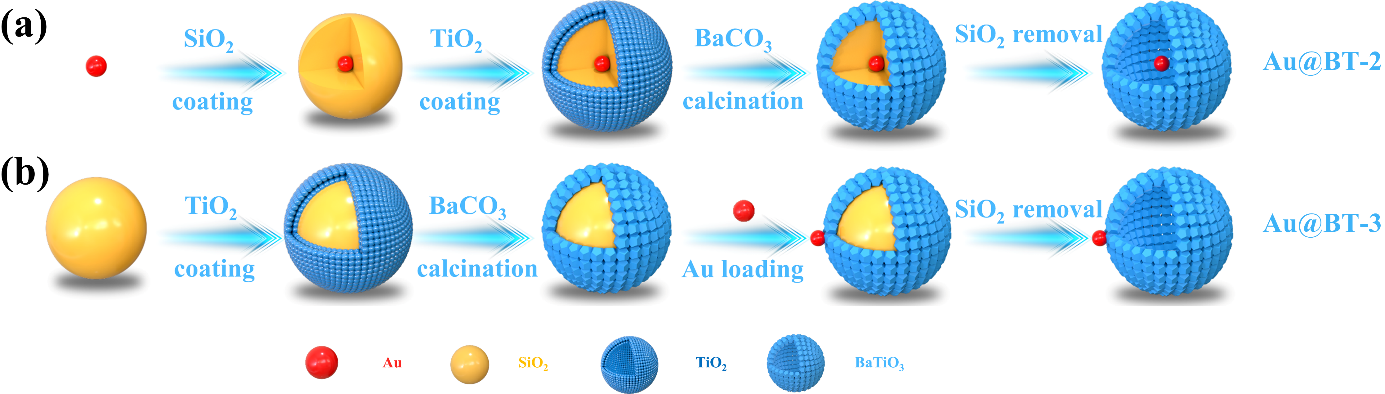


**Figure S1.** Schematic illustration of the fabrication of (a) Au@BT-2 and (b) Au@BT-3.


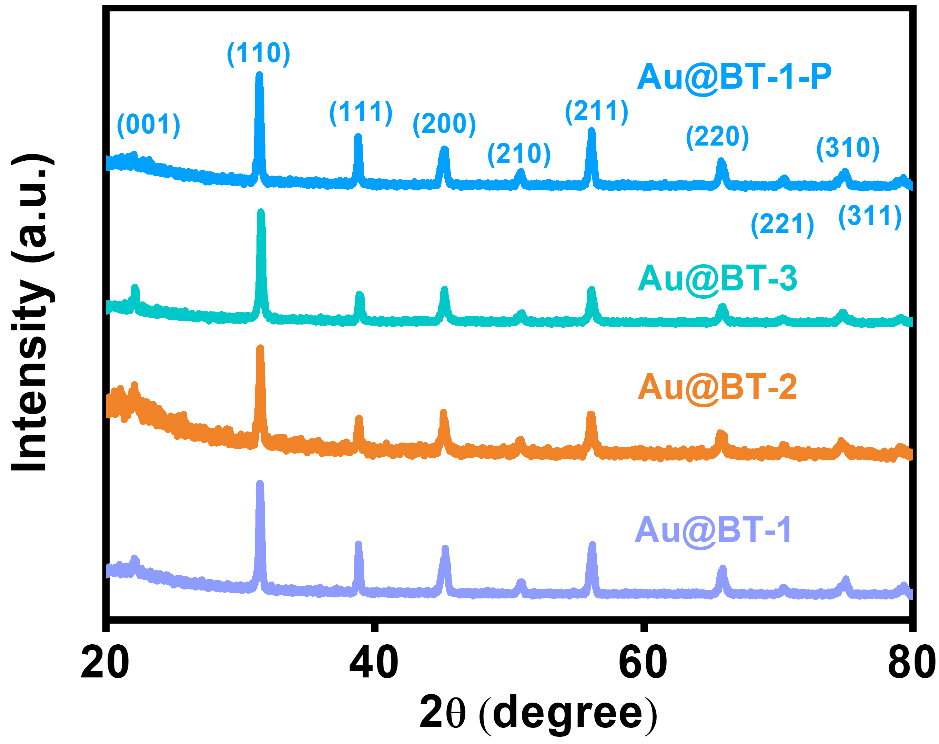


**Figure S2.** XRD patterns of Au@BT-1, Au@BT-2, Au@BT-3, and Au@BT-1-P (JCPDS #79-2263).


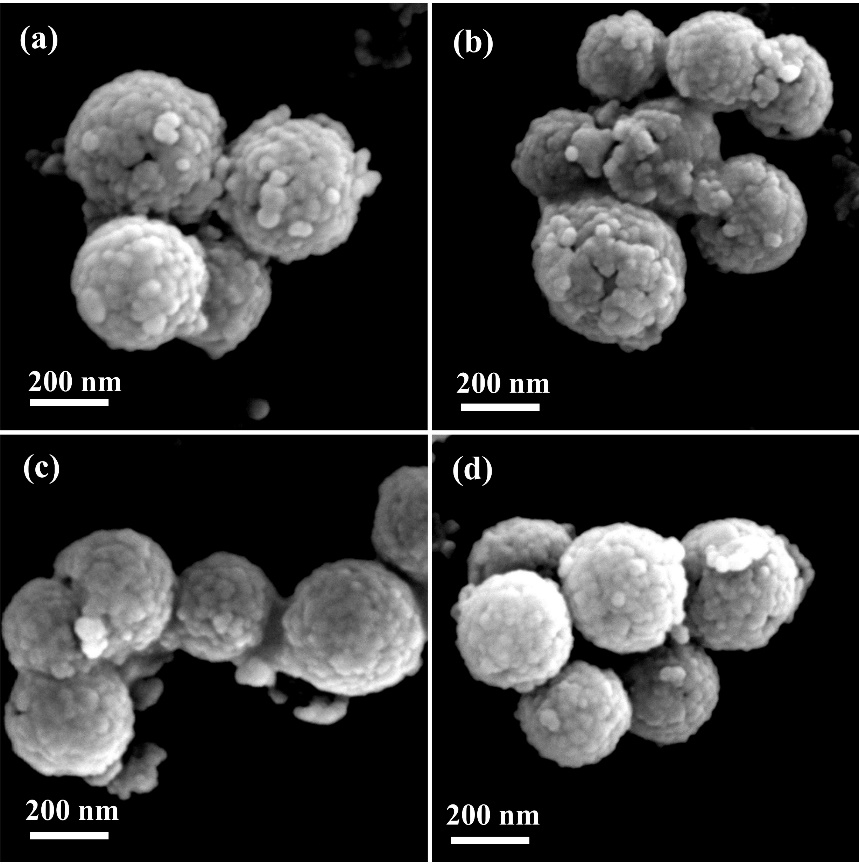


**Figure S3.** (a) SEM images of Au@BT-1, (b) Au@BT-2, (c) Au@BT-3, and (d) Au@BT-1-P.


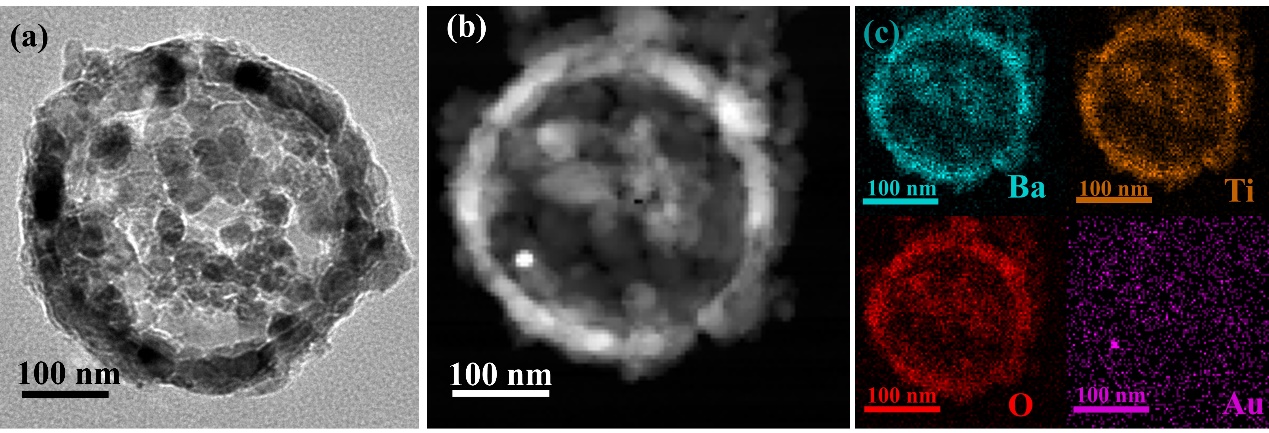


**Figure S4.** (a) TEM image, (b) HAADF-STEM image and (c) EDX maps of Ba, Ti, O, and Au from single Au@BT-2.


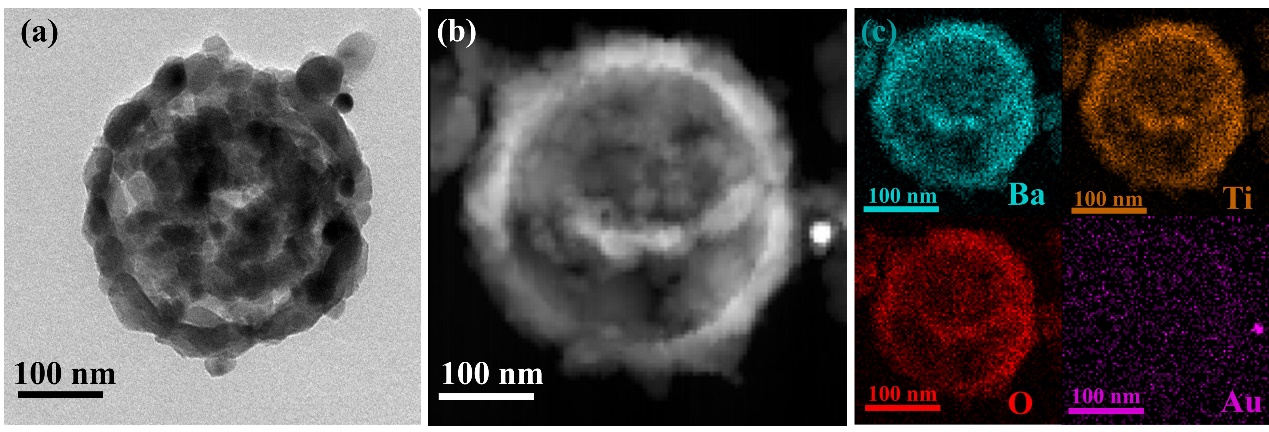


**Figure S5.** (a) TEM image, (b) HAADF-STEM image and (c) EDX maps of Ba, Ti, O, and Au from single Au@BT-3.


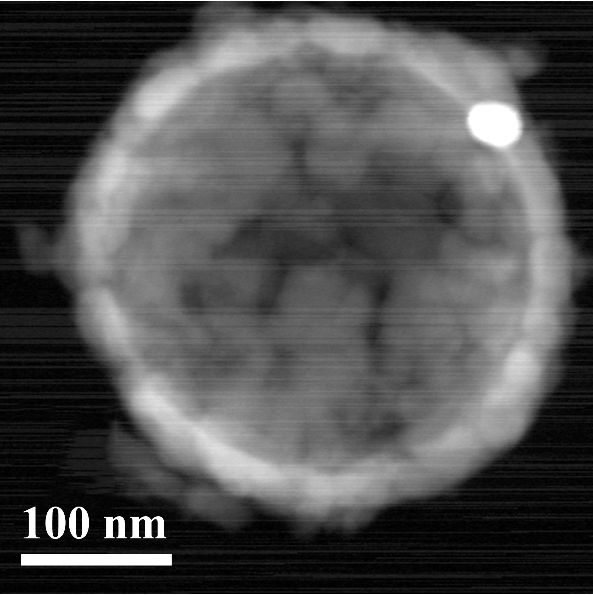


**Figure S6.** HAADF-STEM image of Au@BT-1-P.


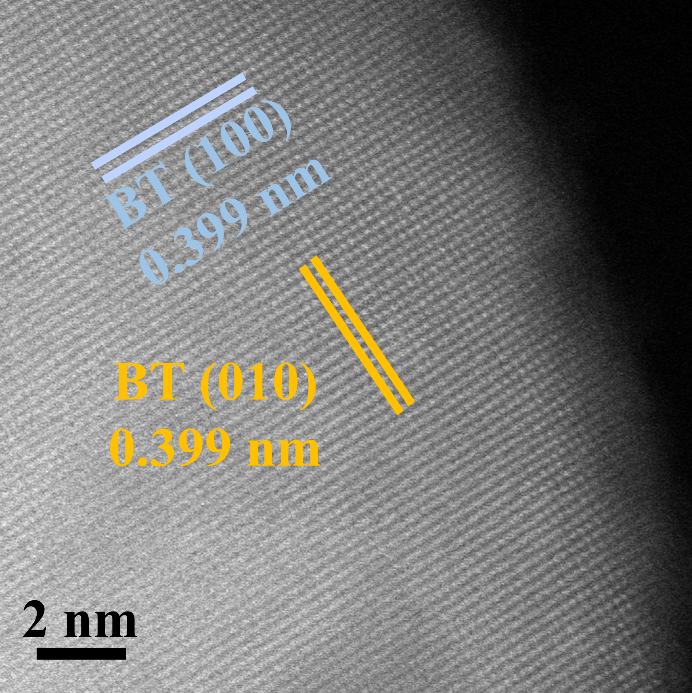


**Figure S7.** HRTEM image of BT in Au@BT-1-P.


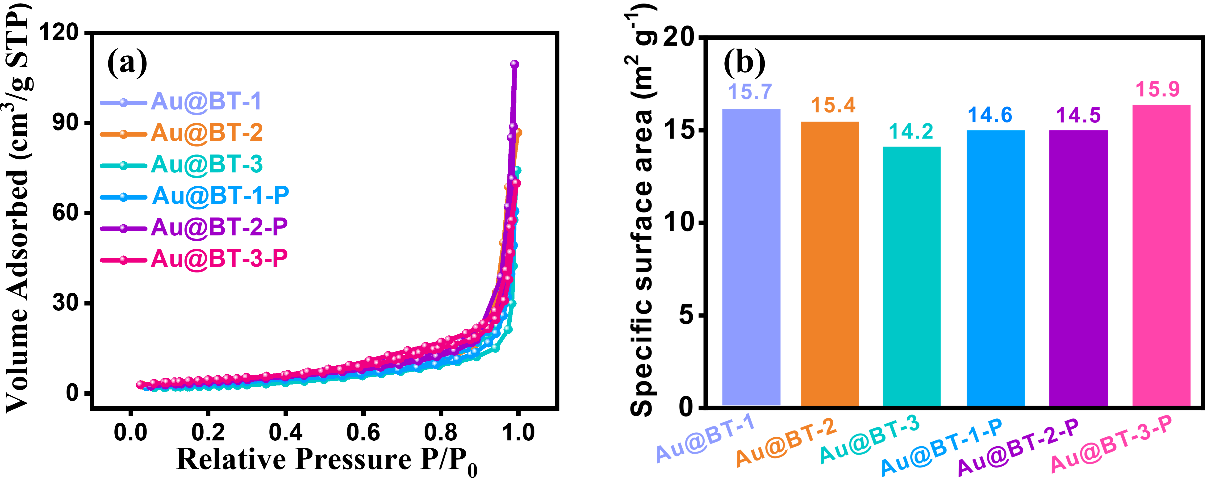


**Figure S8.** (a, b) N_2_ adsorption isotherms and specific surface area of Au@BT-1, Au@BT-2, Au@BT-3, Au@BT-1-P, Au@BT-2-P, and Au@BT-3-P.


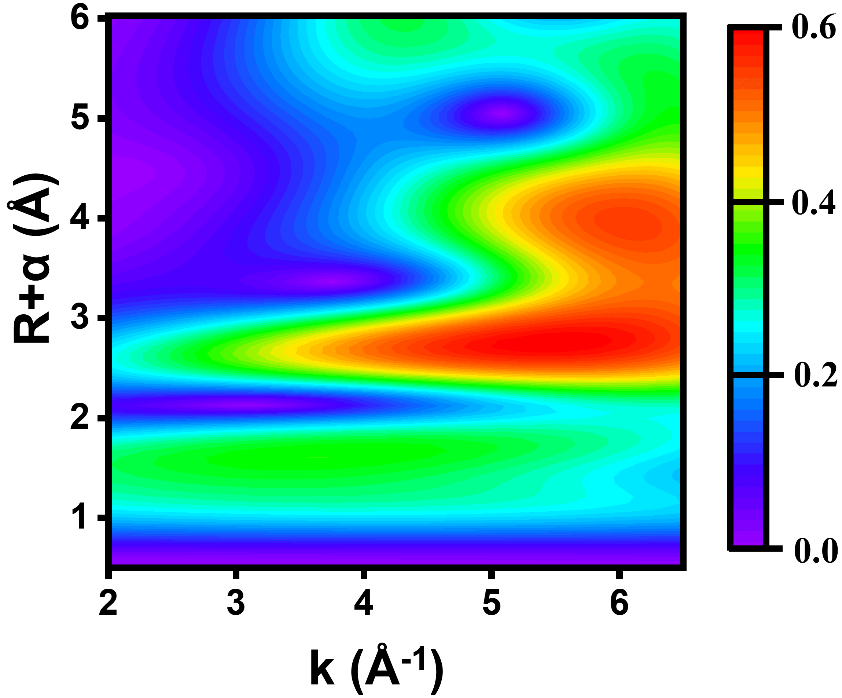


**Figure S9.** WT-EXAFS of BT-HNS.


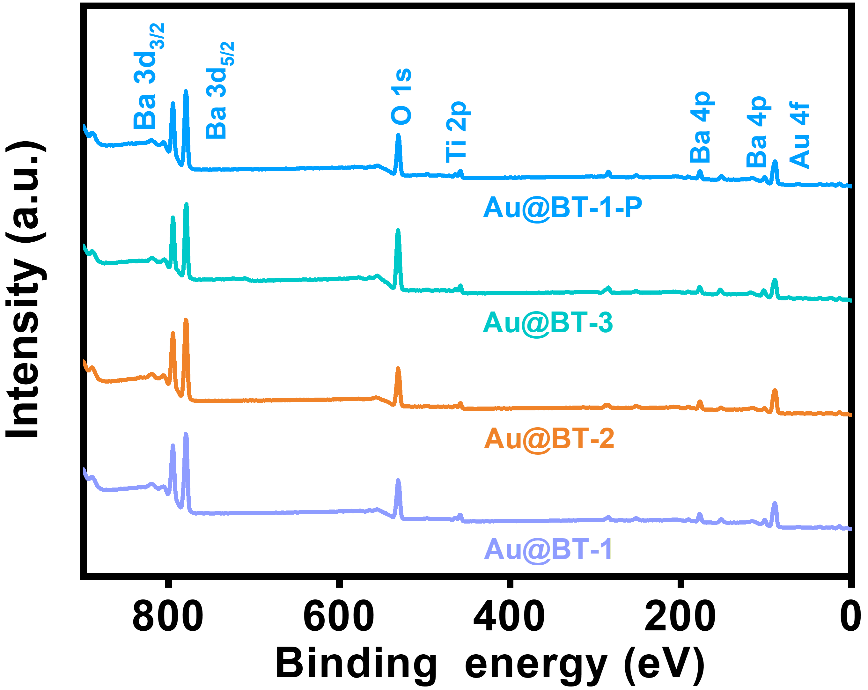


**Figure S10.** XPS survey spectra of Au@BT-1, Au@BT-2, Au@BT-3, and Au@BT-1-P.


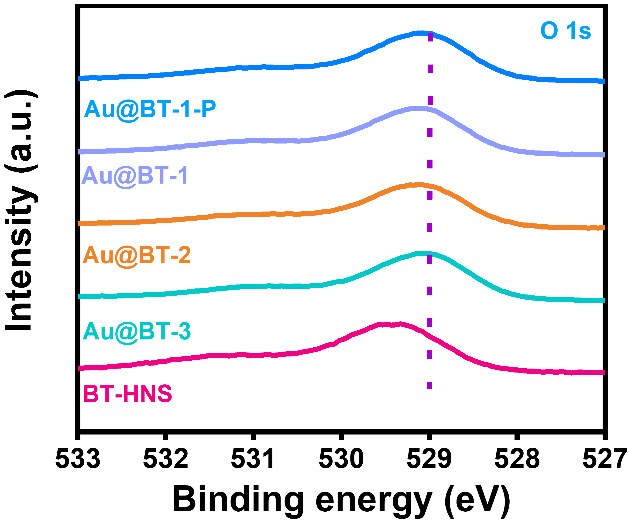


**Figure S11.** XPS spectra of O 1s of BT-HNS, Au@BT-1, Au@BT-2, Au@BT-3, and Au@BT-1-P.


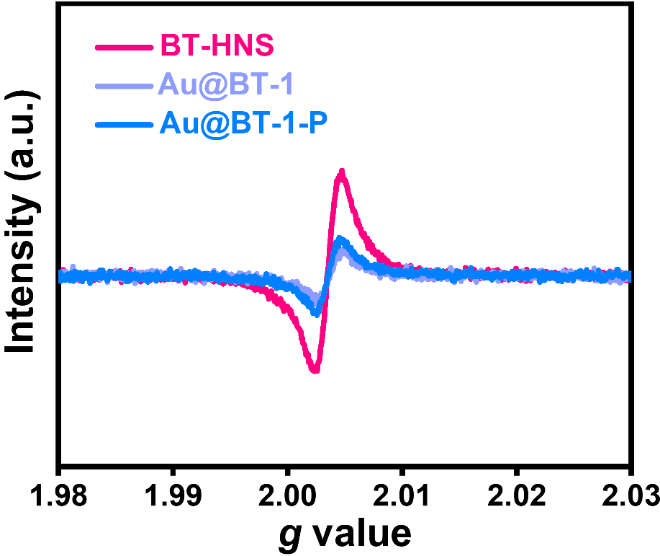


**Figure S12.** EPR plots of BT-HNS, Au@BT-1 and Au@BT-1-P.


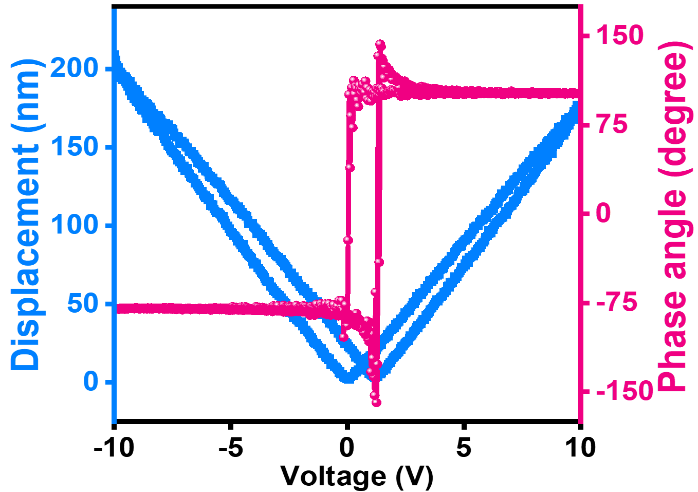


**Figure S13.** The butterfly curve and phase hysteresis loop of Au@BT-1-P.


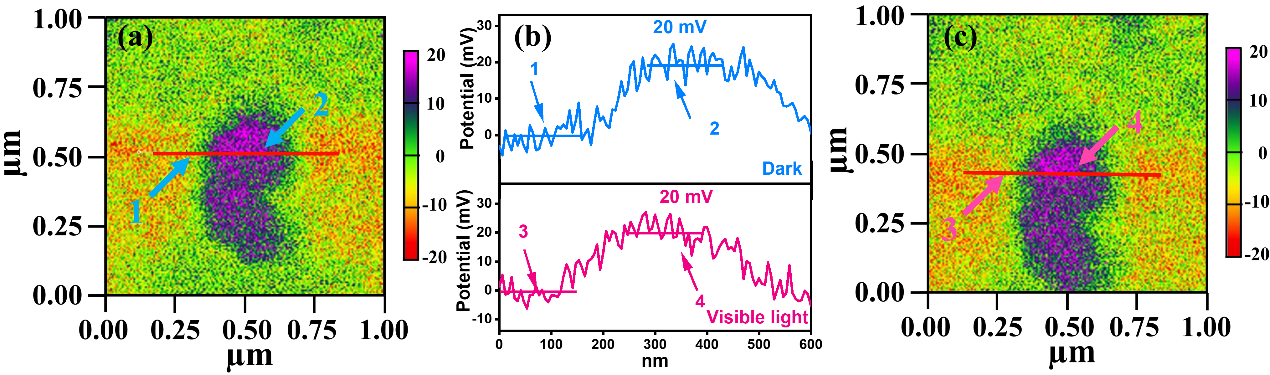


**Figure S14.** (a) Surface charge of BT-HNS in dark, (c) under visible light and (b) corresponding charge difference profile.


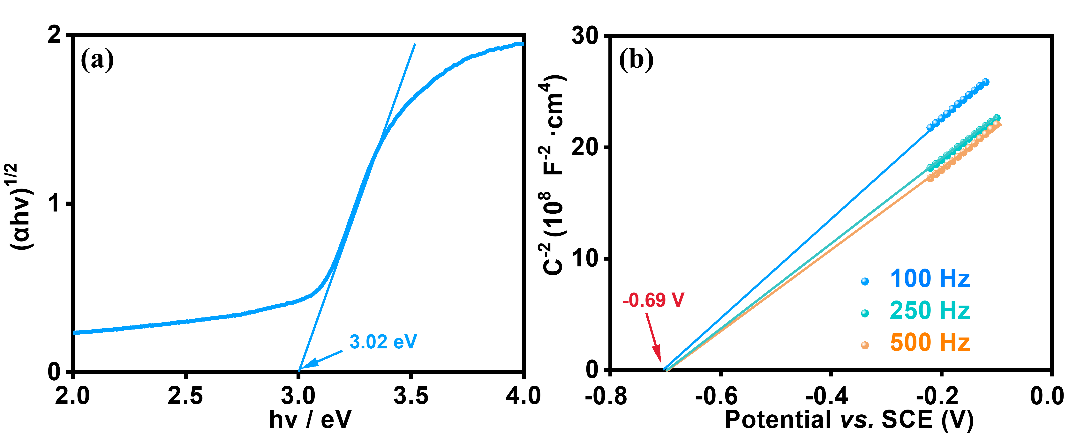


**Figure S15.** (a) Band gap of BT-HNS. (b) Mott-Schottky plots of BT-HNS at frequencies of 100 Hz, 250 Hz and 500 Hz (0.1 M Na_2_SO_4_).


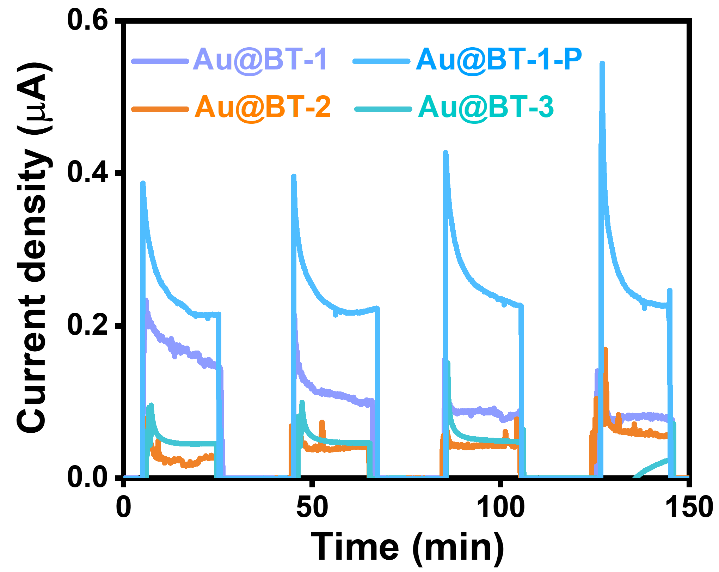


**Figure S16.** Photocurrent responses of Au@BT-1, Au@BT-2, Au@BT-3, and Au@BT-1-P under visible light.


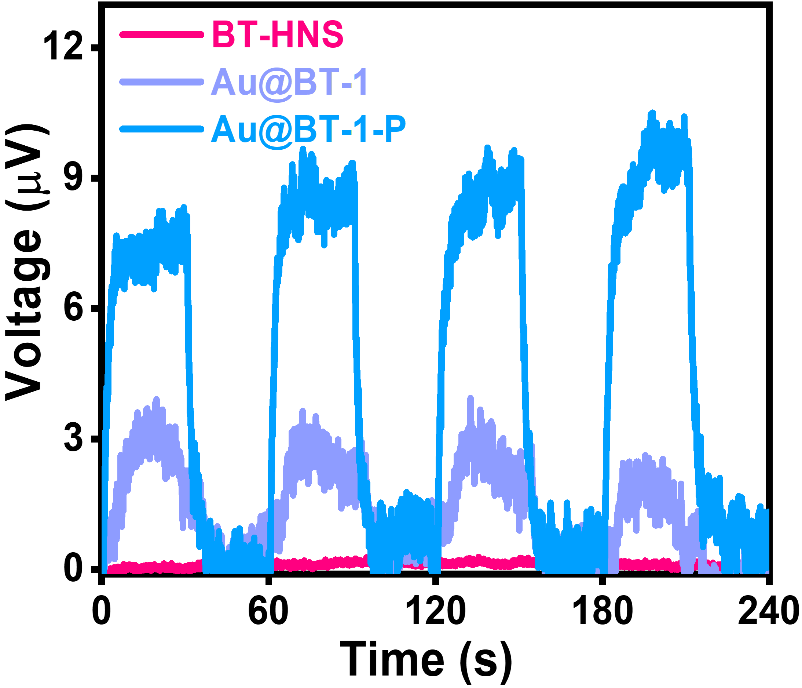


**Figure S17** Open circle voltage of BT-HNS, Au@BT-1, and Au@BT-1-P under the monochromatic light of 550 nm.

**
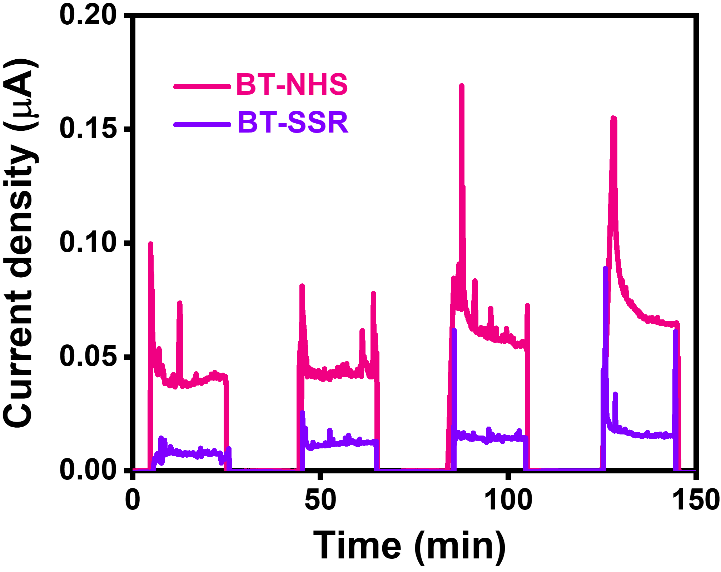
**

**Figure S18.** Piezo-current responses of BT-SSR and BT-NHS under ultrasonic vibration.


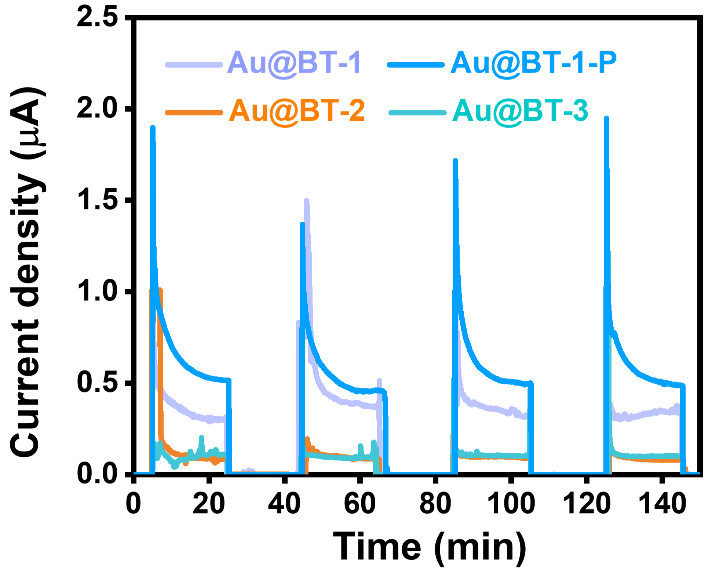


**Figure S19.** Piezo-current responses of Au@BT-1, Au@BT-2, Au@BT-3 and Au@BT-1-P under ultrasonic vibration.

**
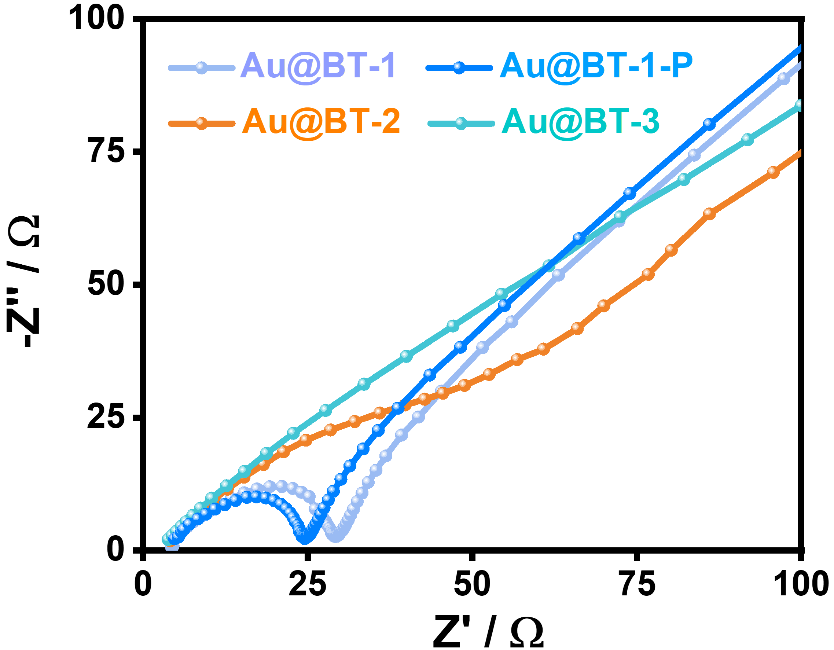
**

**Figure S20.** EIS Nyquist plots of Au@BT-1, Au@BT-2, Au@BT-3 and Au@BT-1-P under ultrasonic vibration and visible light irradiation.


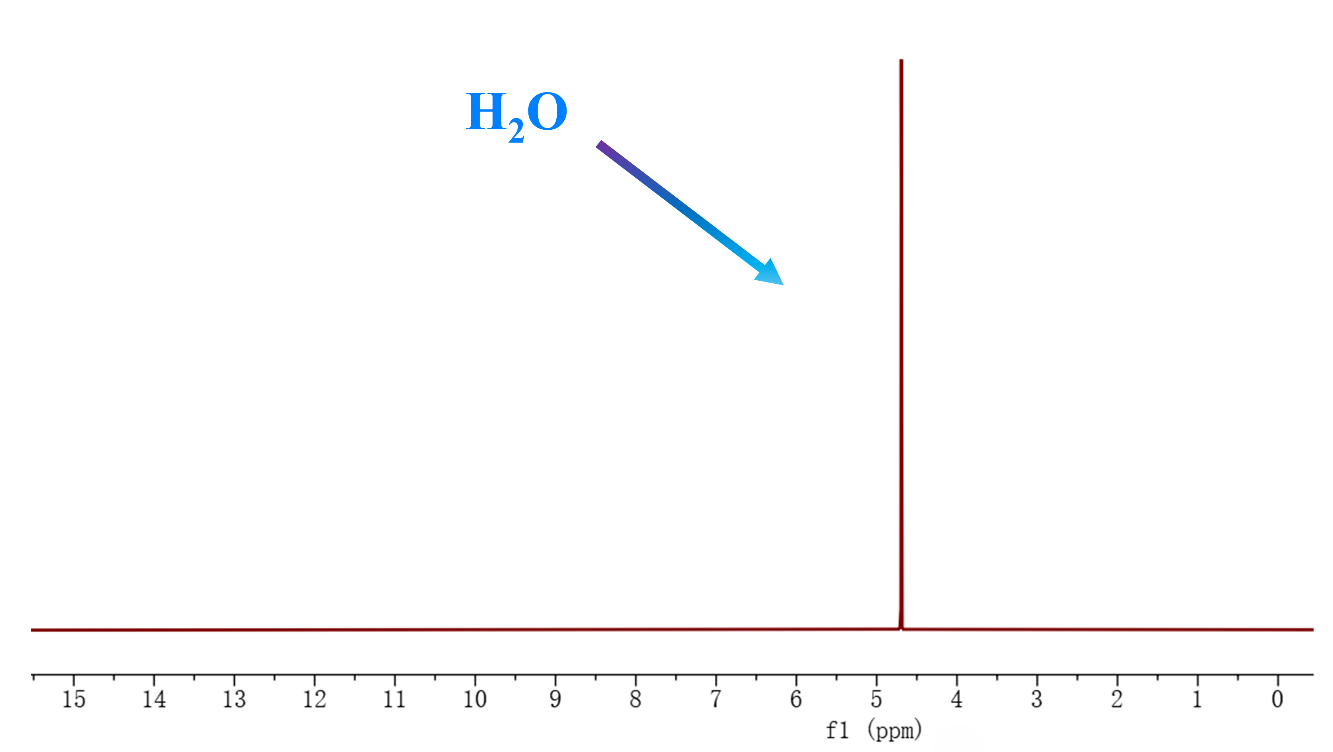


**Figure S21.** ^1^H NMR spectrum of the solution after the catalytic reaction in DMSO.


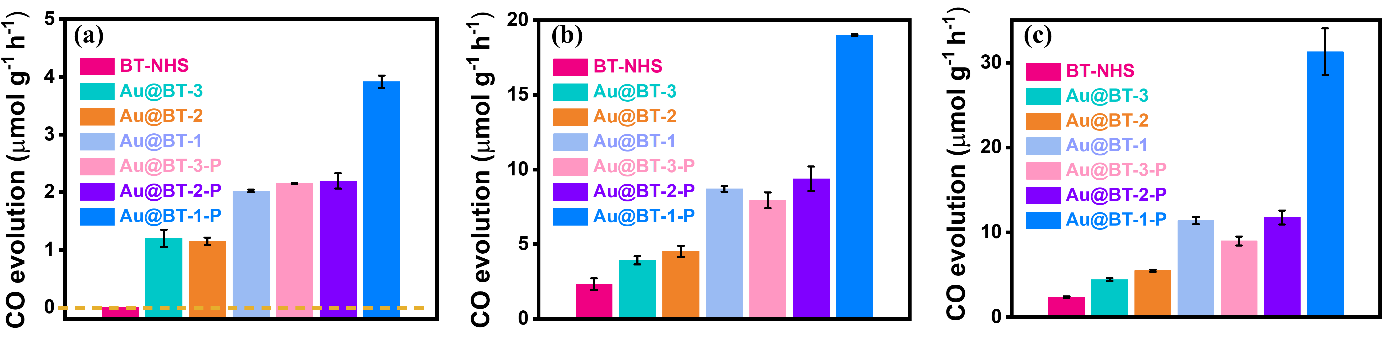


**Figure S22.** (a) Photocatalytic, (b) piezocatalytic and (c) photo-piezocatalytic CO rates over BNT-HNS, Au@BT-2, Au@BT-3, Au@BT-1, Au@BT-2-P, Au@BT-3-P and Au@BT-1-P.


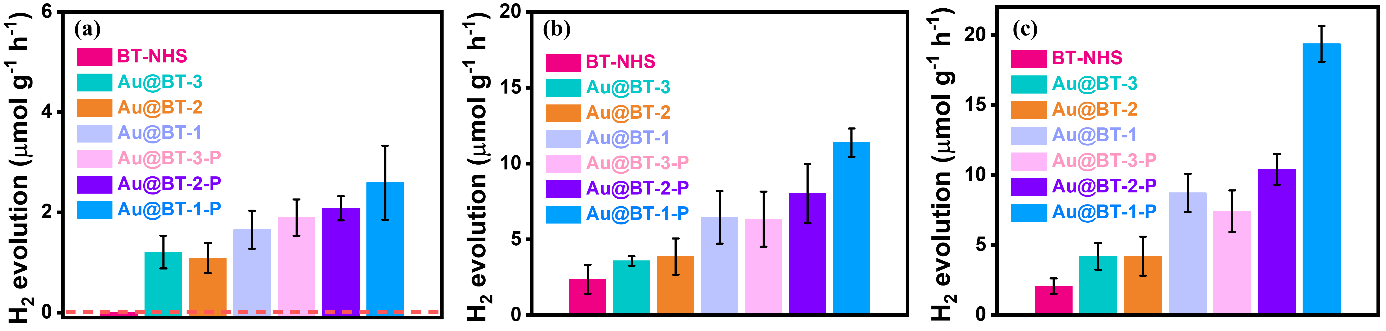


**Figure S23.** (a) Photocatalytic, (b) piezocatalytic and (c) photo-piezocatalytic H_2_ rates over BNT-HNS, Au@BT-2, Au@BT-3, Au@BT-1, Au@BT-2-P, Au@BT-3-P, and Au@BT-1-P.


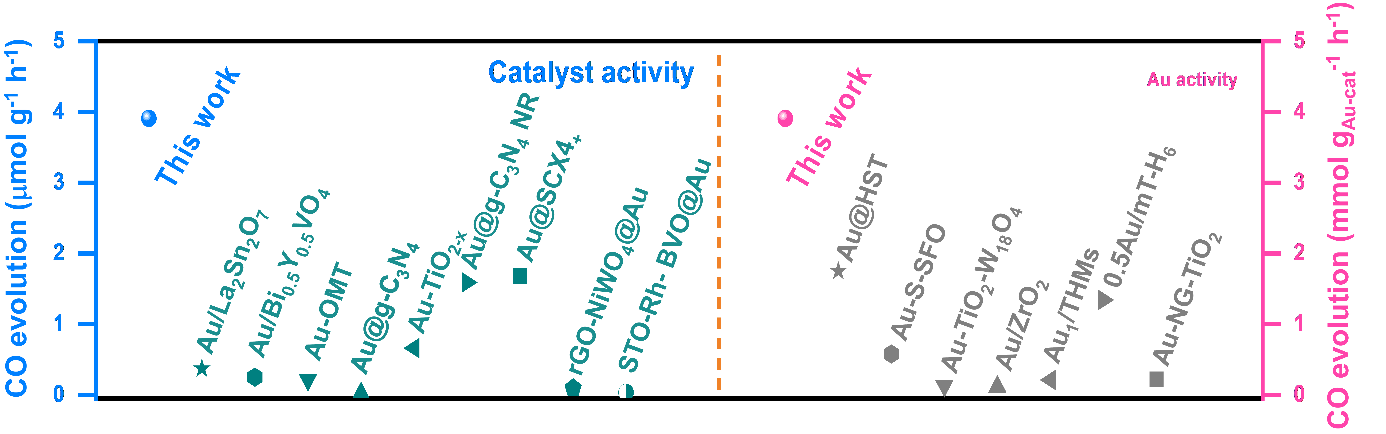


**Figure S24.** Photocatalytic CO evolution performance comparison of Au@BT-1-P with the catalysts reported in literatures (Literatures order 12-27: from left to right). ^[12-27]^


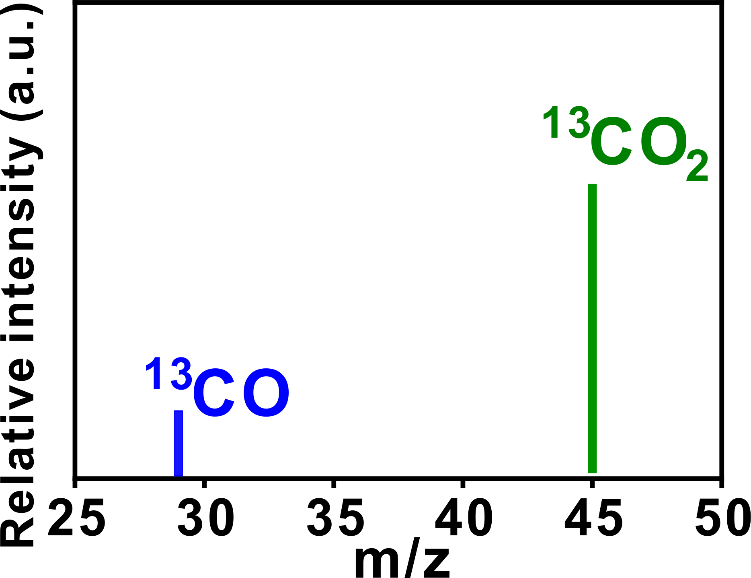


**Figure S25.** MS spectra for photo-piezocatalytic CO_2_ reduction of Au@BT-1-P with using ^13^CO_2_ as the reacting gas.


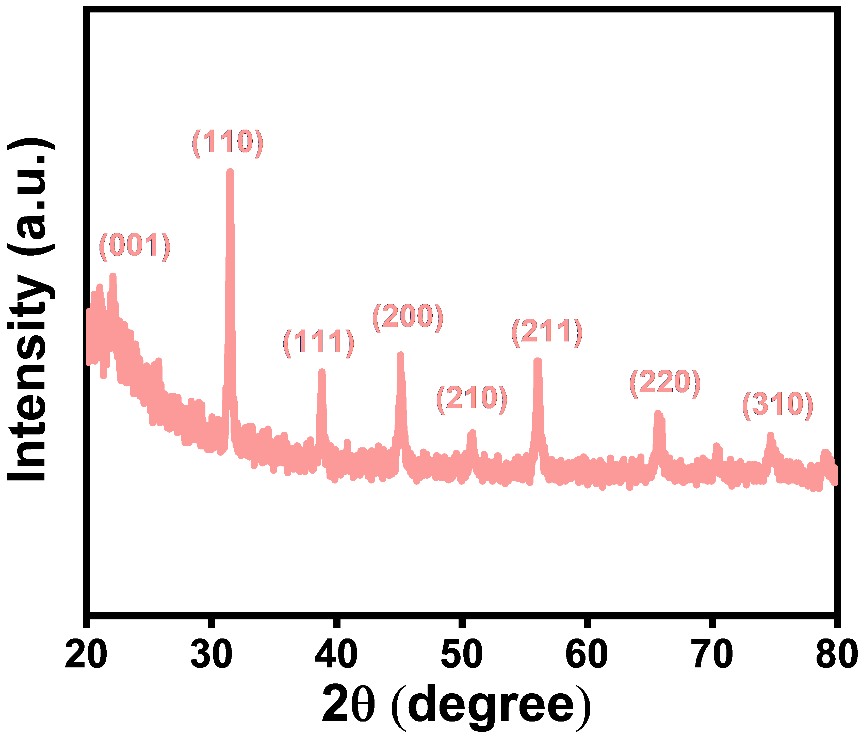


**Figure S26.** XRD pattern of Au@BT-1-P after 20 cycles.


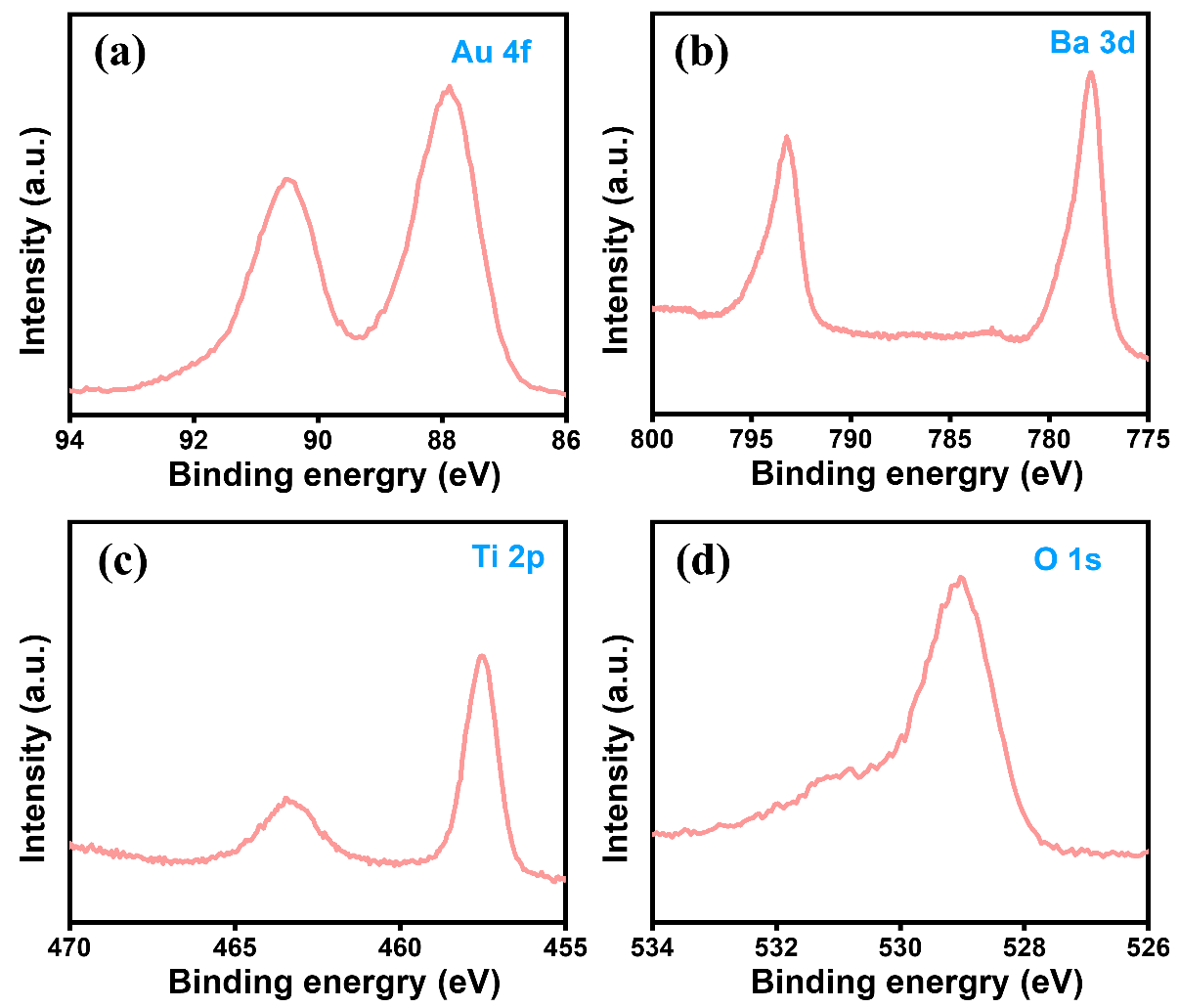


**Figure S27.** XPS spectra of (a) Au 4f, (b) Ba 3d, (c) Ti 2p and (d) O 1s of Au@BT-1-P after 20 cycles.


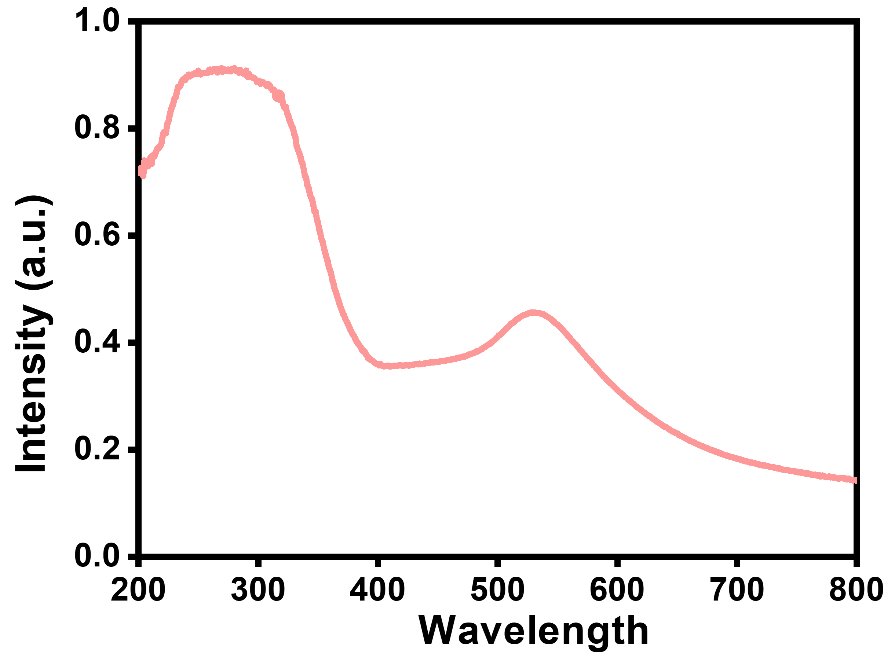


**Figure S28.** UV/Vis diffuse reflectance spectra of Au@BT-1-P after 20 cycles.


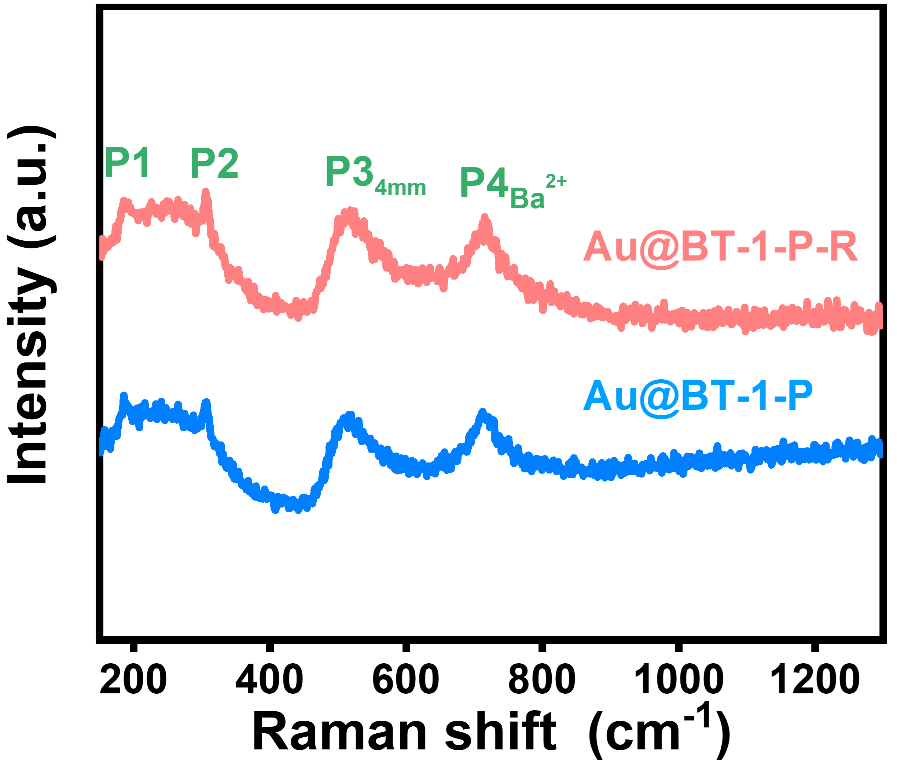


**Figure S29.** Raman spectra of Au@BT-1-P and Au@BT-1-P-R (Au@BT-1-P after 20 cycles).


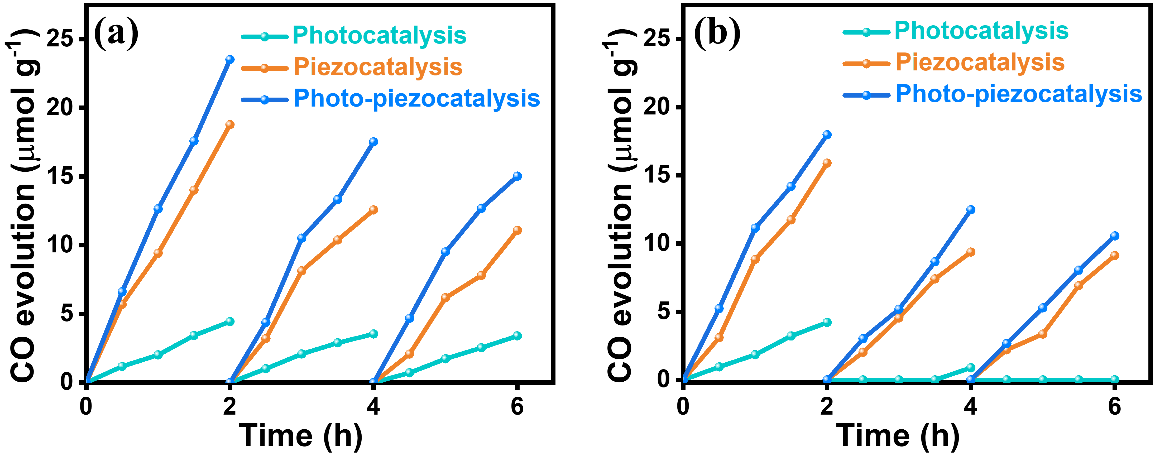


**Figure S30.** CO production through 3 reaction cycles using (a) Au@BT-2-P and (b) Au@BT-3-P under different reaction conditions.


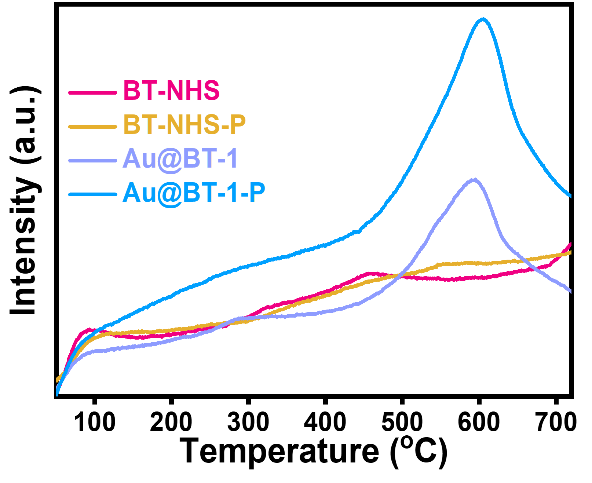


**Figure S31.** CO_2_-TPD profiles of BT-HNS, BT-HNS-P, Au@BT-1, and Au@BT-1-P.


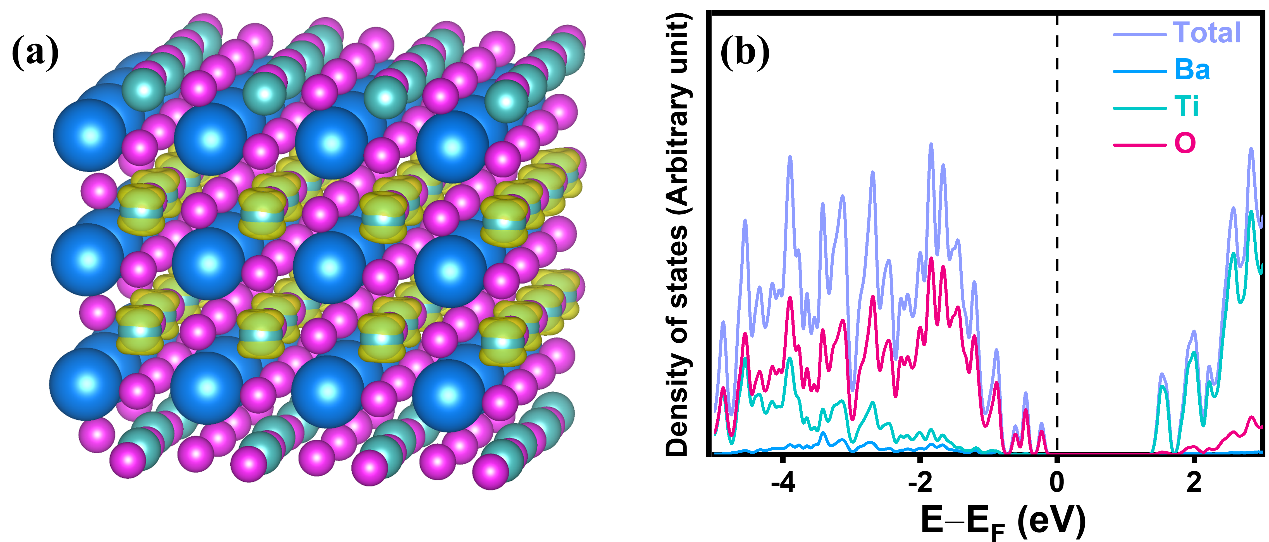


**Figure S32.** (a) The DFT-calculated charge density of the conduction band edge for BaTiO_3_. Ba, Ti, and O atoms are shown in blue, yellow, dark cyan and magenta, respectively. (b) The DFT-calculated PDOSs for BaTiO_3_.


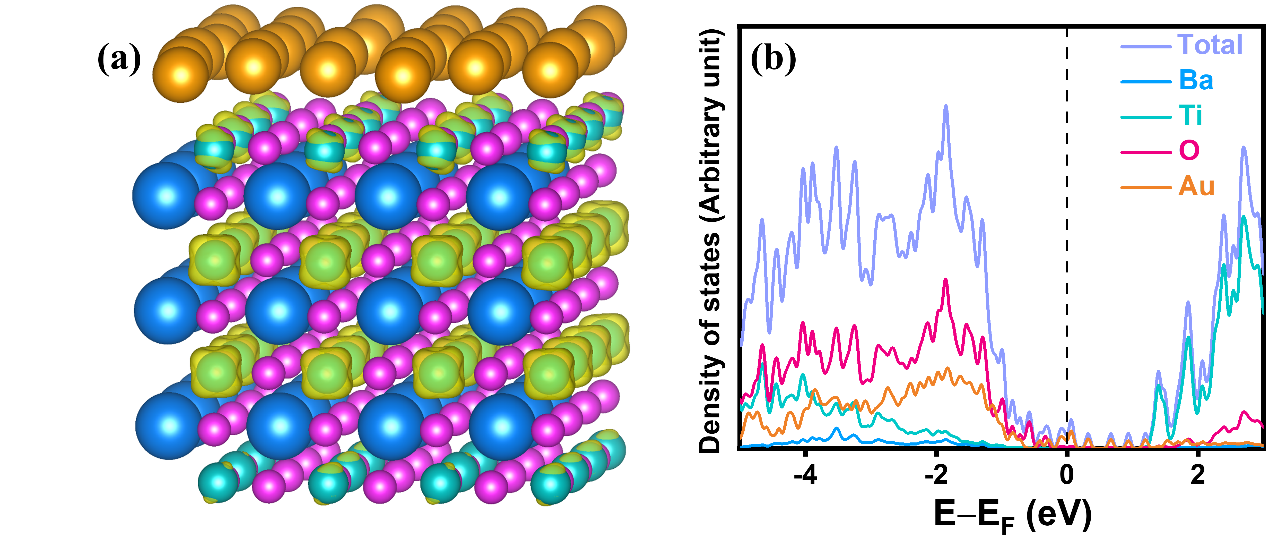


**Figure S33.** (a) The DFT-calculated charge density of the conduction band edge for Au@BaTiO_3_. Ba, Au, Ti, and O atoms are shown in blue, yellow, dark cyan and magenta, respectively. (b) The DFT-calculated PDOSs for Au@BaTiO_3_.


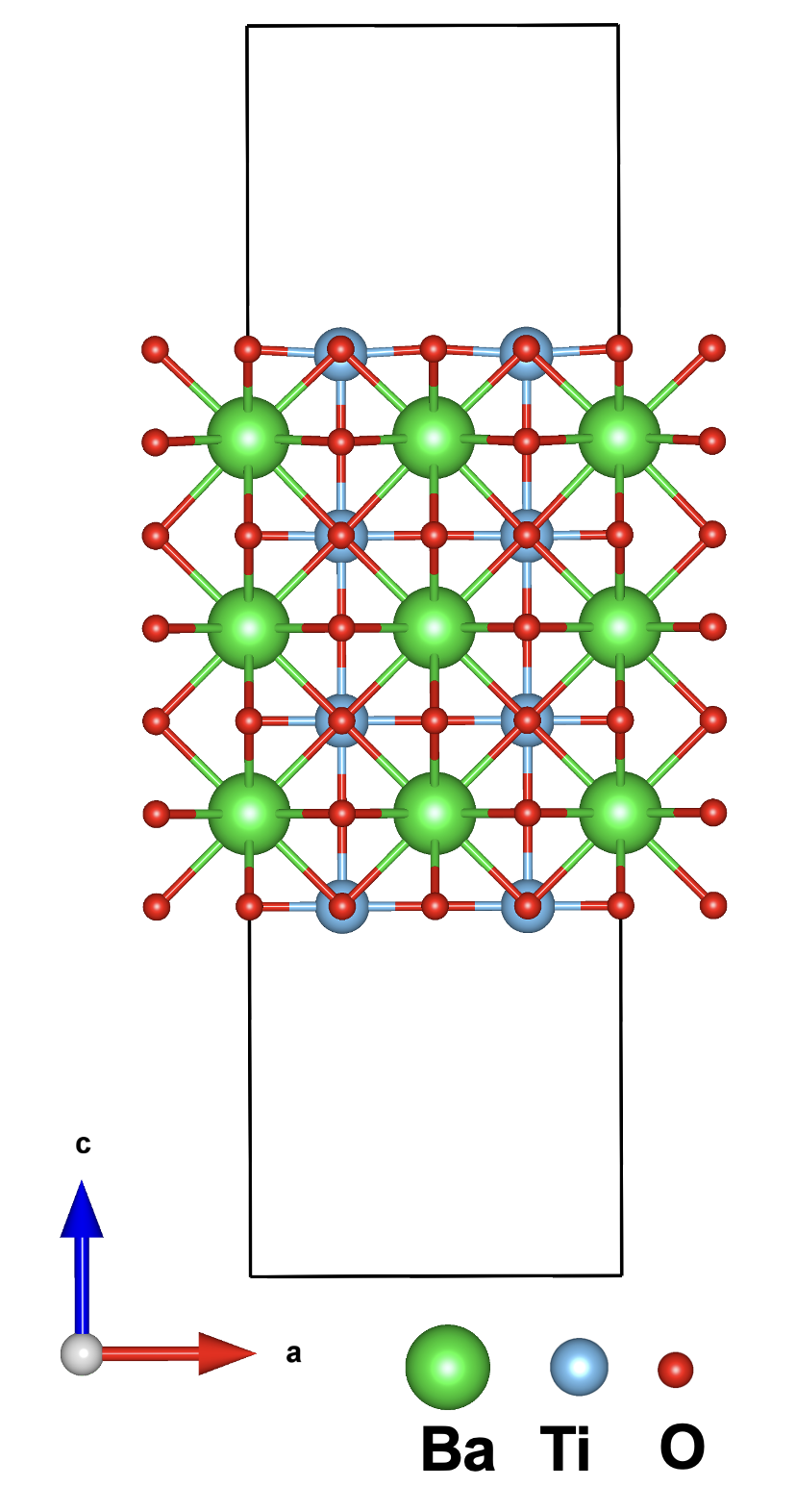


**Figure S34.** Sideview of the supercell of slab model of BaTiO_3_ (001) surface used in the DFT calculations. The Ba, Ti and O atoms are indicated by green, light blue and red spheres, respectively.


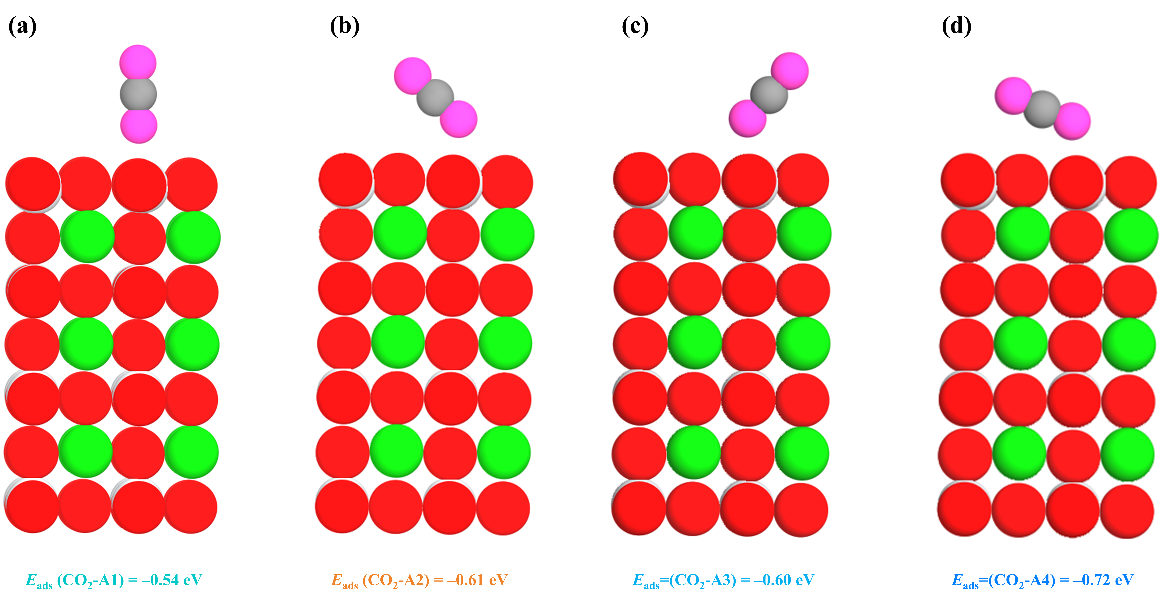


**Figure S35.** Side views of CO_2_ adsorption configuration on fully poled BaTiO_3_ (001) surface. Color code: Ba-green, Ti-white, and O-red in BaTiO_3_, C-gray and O-magenta in CO_2_ molecule.


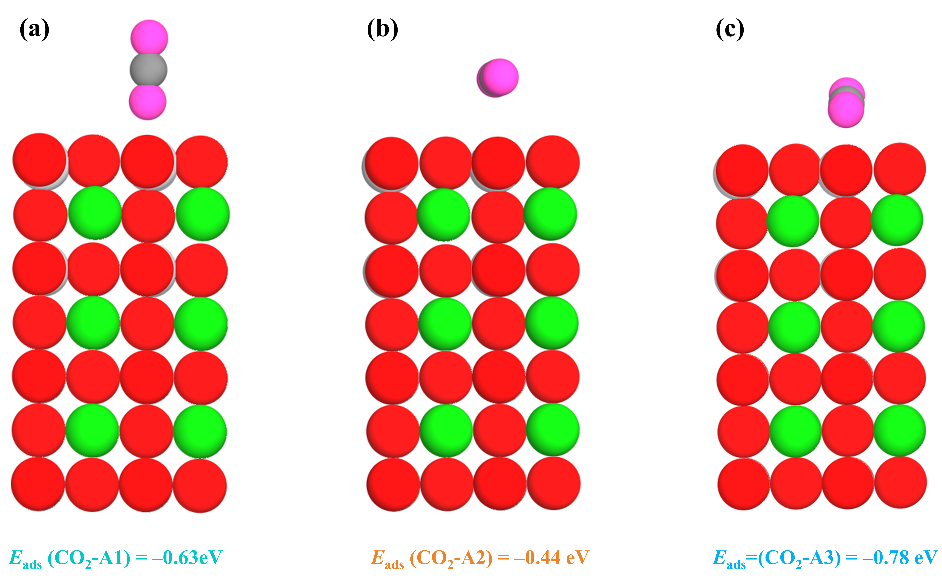


**Figure S36.** Side views of CO_2_ adsorption configuration on un-poled BaTiO_3_ (001) surface. Color code: Ba-green, Ti-white, and O-red in BaTiO_3_, C-gray and O-magenta in CO_2_ molecule.


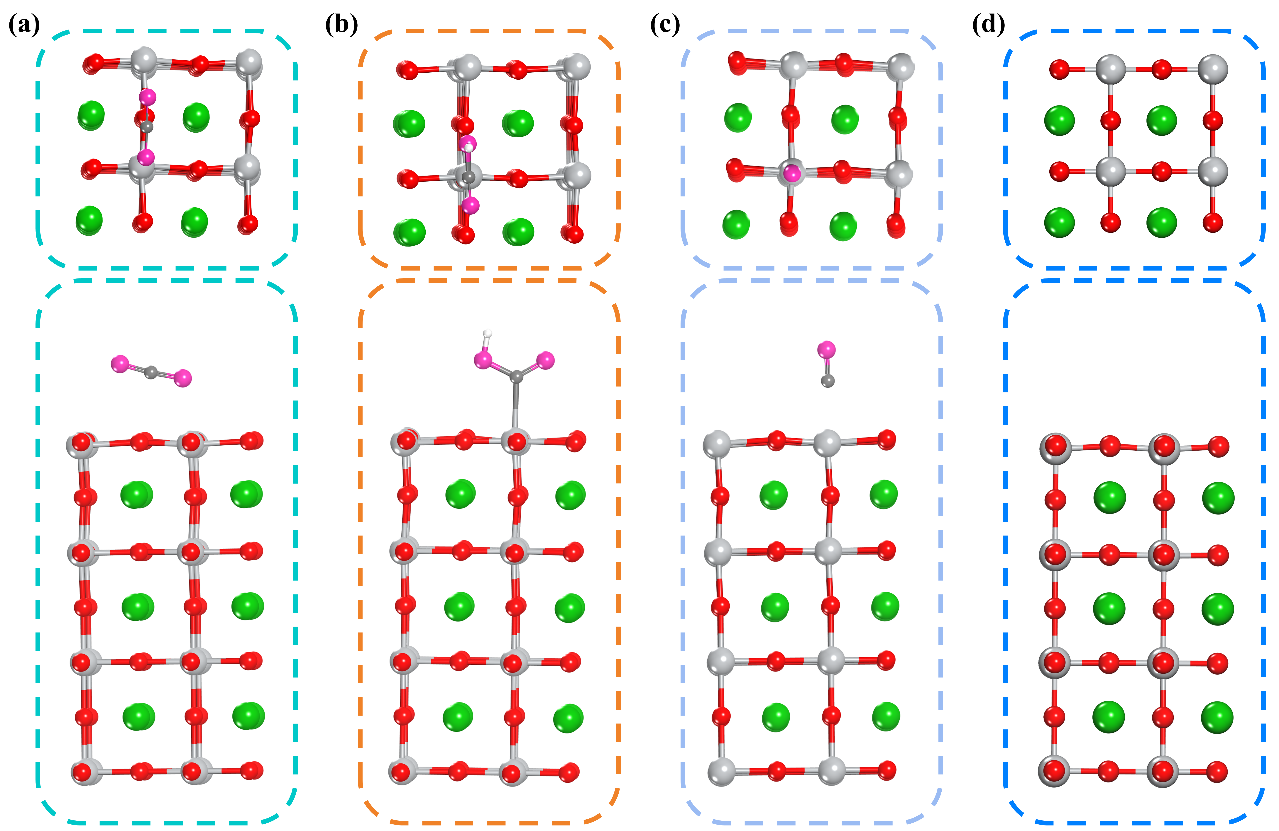


**Figure S37.** Side view and top view of surface species at each step in the process of CO_2_ reduction to CO on un-poled BaTiO_3_ (001) surface. Color code: Ba-green, Ti-grey, O-red, C-black, H-white, O-pink on reactants.


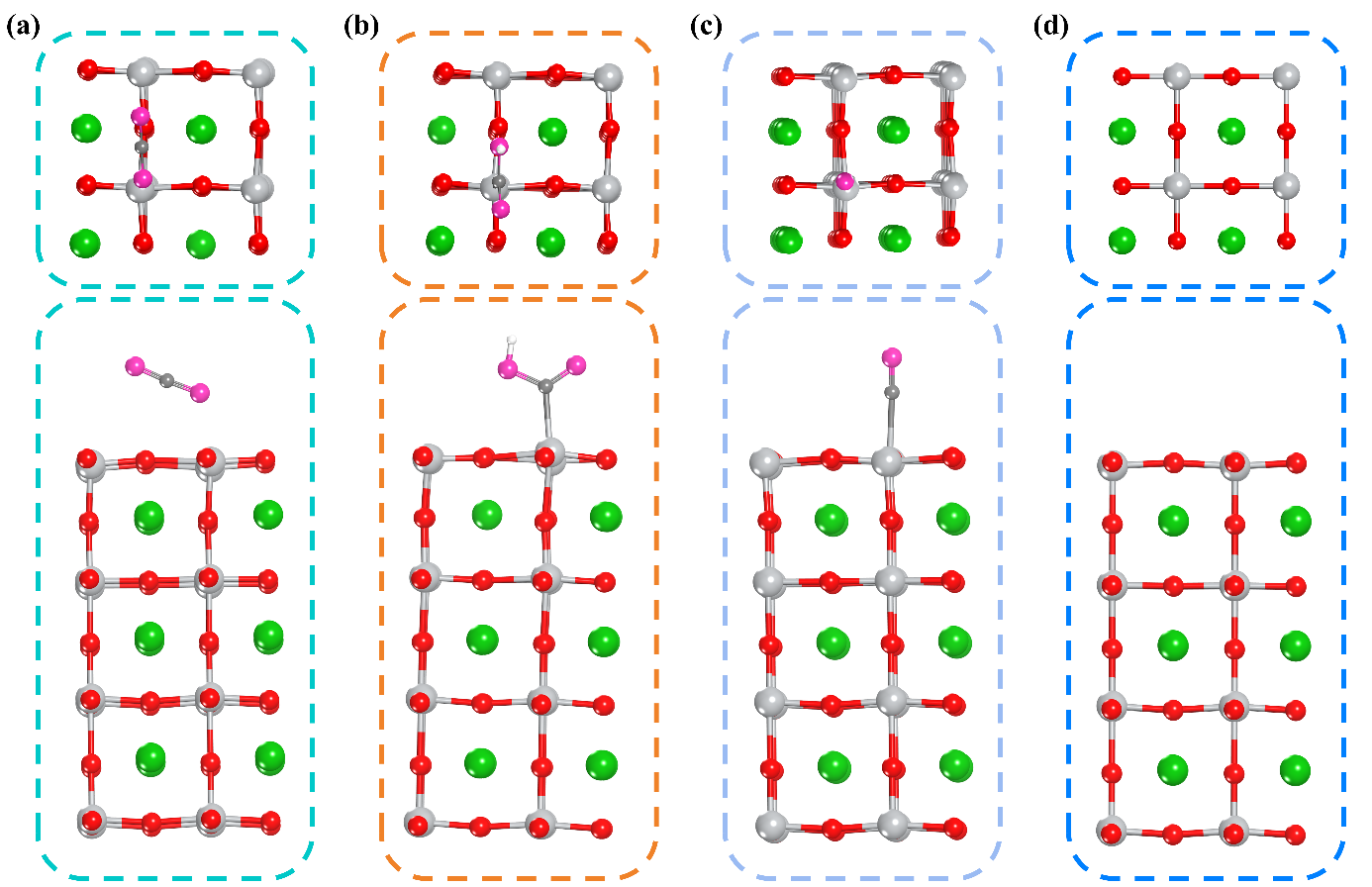


**Figure S38.** Side view and top view of surface species at each step in the process of CO_2_ reduction to CO on poled BaTiO_3_ (001) surface. Color code: Ba-green, Ti-grey, O-red, C-black, H-white, O-pink on reactants.


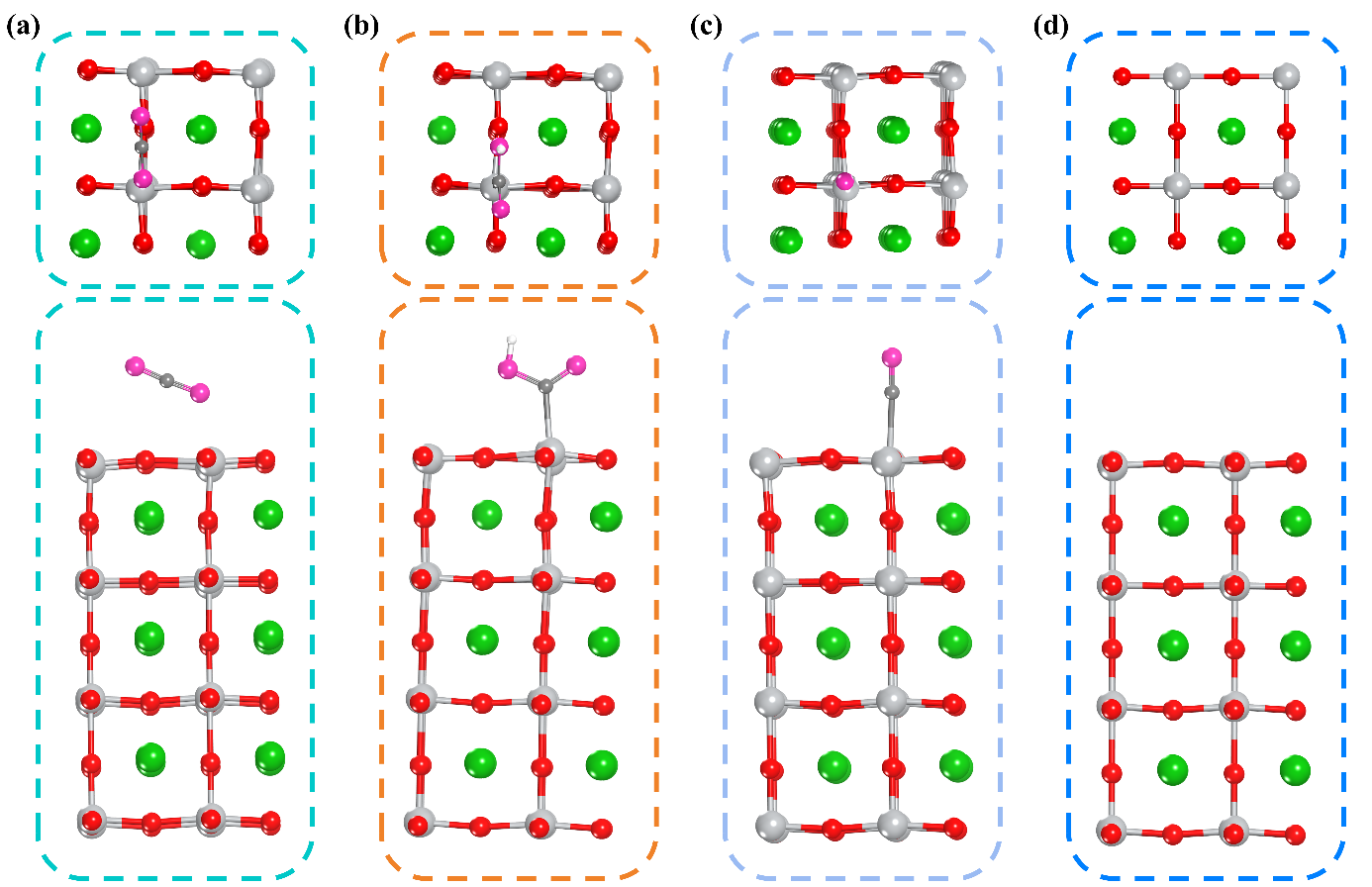


**Figure S39.** Side view and top view of surface species at each step in the process of CO_2_ reduction to CO on poled BaTiO_3_ (001) surface with extra pressure. Color code: Ba-green, Ti-grey, O-red, C-black, H-white, O-pink on reactants.

**3. Supplementary Tables**

**Table S1.** Specific surface area and pore diameter of different catalysts.

| **Samples** | **Specific surface area (m^2^ g^-1^)** | **Pore diameter (nm)** |
| --- | --- | --- |
| Au@BT-1 | 15.7 | 24.4 |
| Au@BT-2 | 14.2 | 23.2 |
| Au@BT-3 | 15.4 | 26.3 |
| Au@BT-1-P | 15.9 | 23.9 |
| Au@BT-2-P | 14.6 | 25.7 |
| Au@BT-3-P | 14.5 | 28.3 |
| BT-SSR | 0.2 | 243.5 |

**T****able S2.** Time resolved PL decay parameter for Au@BT-1, Au@BT-2, Au@BT-3 and Au@BT-1-P.

| **Samples** | **a_1_ (%)** | ***τ*_1_ (ns)** | **a_2_ (%)** | ***τ*_2_ (ns)** | **τ_ave_ (ns)** |
| --- | --- | --- | --- | --- | --- |
| Au@BT-1 | 75.55 | 0.48 | 24.45 | 3.97 | 1.34 |
| Au@BT-2 | 77.08 | 0.61 | 22.92 | 3.42 | 1.25 |
| Au@BT-3 | 84.89 | 0.46 | 15.11 | 5.07 | 1.15 |
| Au@BT-1-P | 70.81 | 0.51 | 29.19 | 5.04 | 1.83 |

**Table S3.** Photocatalytic, piezocatalytic and photo-piezocatalytic CO rates of BT-SSR, BT-NHS, Au@BT-1, Au@BT-2, Au@BT-3, Au@BT-1-P, Au@BT-2-P, and Au@BT-3-P.

| **Samples** | **Photocatalysis**  **(μmol g^-1^ h^-1^)** | **Piezocatalysis**  **(μmol g^-1^ h^-1^)** | **Photo-piezocatalysis**  **(μmol g^-1^ h^-1^)** |
| --- | --- | --- | --- |
| BT-SSR | 0 | 0.15 | 0.14 |
| BT-NHS | 0 | 2.33 | 2.94 |
| Au@BT-3 | 1.2 | 3.93 | 4.43 |
| Au@BT-2 | 1.15 | 4.53 | 5.48 |
| Au@BT-1 | 2.02 | 8.71 | 11.38 |
| Au@BT-3-P | 2.15 | 7.95 | 8.98 |
| Au@BT-2-P | 2.20 | 9.38 | 11.75 |
| Au@BT-1-P | 3.92 | 19.02 | 31.29 |

**Table S4.** Photocatalytic, piezocatalytic and photo-piezocatalytic H_2_ rates of BT-NHS, Au@BT-1, Au@BT-2, Au@BT-3, Au@BT-1-P, Au@BT-2-P and Au@BT-3-P.

| **Samples** | **Photocatalysis**  **(μmol g^-1^ h^-1^)** | **Piezocatalysis**  **(μmol g^-1^ h^-1^)** | **Photo-piezocatalysis**  **(μmol g^-1^ h^-1^)** |
| --- | --- | --- | --- |
| BT-SSR | 0 | 0.11 | 0.09 |
| BT-NHS | 0 | 2.38 | 2.06 |
| Au@BT-3 | 1.21 | 3.58 | 4.18 |
| Au@BT-2 | 1.09 | 3.87 | 4.20 |
| Au@BT-1 | 1.65 | 6.45 | 8.73 |
| Au@BT-3-P | 1.90 | 6.34 | 7.42 |
| Au@BT-2-P | 2.08 | 8.02 | 10.41 |
| Au@BT-1-P | 2.59 | 11.38 | 19.38 |

**Reference**

[1] D. Kuo, C. Chang, T. Su, W. Wang, B. Lin, *J. Eur. Ceram. Soc.* **2001**, *21*, 1171-1177.

[2] N. Horchidan, L. Padurariu, C. Ciomaga, L. Curecheriu, M. Airimioaei, F. Doroftei, F. Tufescu, L. Mitoseriu, *J. Eur. Ceram. Soc.* **2020**, *40*, 1258-1268.

[3] G. Kresse, J. Hafner, *Phys. Rev. B* **1994**, *49*, 14251-14269.

[4] J. Perdew, K. Burke, M. Ernzerhof, *Phys. Rev. Lett.* **1996**, *77*, 3865-3868.

[5] P. Blöchl, *Phys. Rev. B* **1994**, *50*, 17953.

[6] P. Abbasi, M. Barone, M. Cruz-Jáuregui, D. Valdespino-Padilla, H. Paik, T. Kim, L. Kornblum, D. Schlom, T. Pascal, D. Fenning, *Nano Lett.* **2022**, *22*, 4276-4284.

[7] H. Yu, F. Chen, X. Li, H. Huang, Q. Zhang, S. Su, K. Wang, E. Mao, B. Mei, G. Mul, *Nat. Commun.* **2021,** *12*, 1-10.

[8] S. Grimme, J. Antony, S. Ehrlich, H. Krieg, *J. Chem. Phys.* **2010**, *132*, 154104.

[9] H. Monkhorst, J. Pack, *Phys. Rev. B* **1976**, *13*, 5188-5192.

[10] J. Ma, S. Jing, Y. Wang, X. Liu, L. Gan, C. Wang, J. Dai, X. Han, X. Zhou, *Adv. Energy Mater.* **2022**, *12*, 2200253.

[11] R. Buttner, E. Maslen, *Acta Crystallogr., Sect. B: Struct. Sci.* **1992**, *48*, 764-769.

[12] M. Gu, D. Liu, T. Ding, X. Liu, T. Chen, X. Shen, T. Yao, *Dalton Trans*. **2021,** *50*, 6076-6082.

[13] S. Chen, B. Pan, L. Zeng, S. Luo, X. Wang, W. Su, *RSC adv.* **2017,** *7*, 14186-14191.

[14] W. Chen, Y. Wang, W. Shangguan, *Mater. Lett.* **2019,** *238*, 74-76.

[15] X. Liu, M. Ye, S. Zhang, G. Huang, C. Li, J. Yu, P.K. Wong, S. Liu, *J. Mater. Chem. A* **2018**, *6*, 24245-24255.

[16] S. Cai, J. Chen, Q. Li, H. Jia, *ACS Appl. Mater. Interfaces* **2021,** *13*, 14221-14229.

[17] H. Xue, T. Wang, H. Gong, H. Guo, X. Fan, B. Gao, Y. Feng, X. Meng, X. Huang, J. He, *Chem-Asian J.* **2018,** *13*, 577-583.

[18] J. Tian, K. Zhong, X. Zhu, J. Yang, Z. Mo, J. Liu, J. Dai, Y. She, Y. Song, H. Li, *Chem. Eng. J.* **2023,** *451*, 138392.

[19] T. Yoshii, K. Tamaki, Y. Kuwahara, K. Mori, H. Yamashita, *J. CO_2_ Util.* **2021,** *52*, 101691.

[20] W. Chen, Y. Wang, W. Shangguan, *Int. J. Hydrogen Energ.* **2019,** *44*, 4123-4132.

[21] J. Shin, J. Heo, J.Y. Do, Y. Kim, S. Yoon, Y. Kim, M. Kang, *J. Ind. Eng. Chem.* **2020**, *81*, 427-439.

[22] X. Jiang, J. Huang, Z. Bi, W. Ni, G. Gurzadyan, Y. Zhu, Z. Zhang, *Adv. Mater.* **2022**, *34*, 2109330.

[23] T. Skorjanc, K. Kamal, A. Alkhoori, G. Mali, A. Mohammed, Z. Asfari, K. Polychronopoulou, B. Likozar, A. Trabolsi, D. Shetty, *ACS Appl. Mater. Interfaces* **2022**, *14*, 30796-30801.

[24] S. Li, N. Hasan, H. Ma, O. Li, B. Lee, Y. Jia, C. Liu, *Sep. Purif. Technol.* **2022,** *299*, 121650.

[25] K. Wang, J. Lu, Y. Lu, C.H. Lau, Y. Zheng, X. Fan, *Appl. Catal. B: Environ.* **2021,** *292*, 120147.

[26] S. Yoshino, K. Sato, Y. Yamaguchi, A. Iwase, A. Kudo, *ACS Appl.Energ. Mater.* **2020,** *3*, 10001-10007.

[27] D. Wang, Y. Li, B. Yu, H. Li, W. Jiang, X. Deng, Y. Wen, C. Liu, G. Che, *Adv. Powder Technol.* **2021,** *32*, 1653-1662.
